# Supplementary material for: The Advanced Floating Chirality Distance Geometry Approach―How Anisotropic NMR Parameters Can Support the Determination of the Relative Configuration of Natural Products
Source: Mar Drugs. 2020 Jun 24;18(6):330. doi: 10.3390/md18060330 (PMC7344786; doi:10.3390/md18060330)
Supplement: Supplementary file 1 [file marinedrugs-18-00330-s001.pdf]

## Supporting Information for

# The advanced floating chirality distance geometry approach – how anisotropic NMR parameters can support the determination of the relative configuration of natural products

Matthias Köck[a], Michael Reggelin[b], and Stefan Immel[b]

[a] Alfred-Wegener-Institut für Polar- und Meeresforschung in der Helmholtz-Gemeinschaft, Am Handelshafen 12, 27570 Bremerhaven, Germany

[b] Clemens-Schöpf-Institut für Organische Chemie und Biochemie, Technische Universität Darmstadt, Alarich-Weiss-Straße 4, 64287 Darmstadt, Germany

## Table of Contents

Formulas and Atom Numbering of Compounds (1)-(5) 1
NOE and Structure Data for Axinellamine A (1) 2
NOE and Structure Data for Tetrabromostyloguanidine (2) 4
NOE and Structure Data for 3,7-*epi*-Massadine chloride (3) 8
NOE, RDC, and Structure Data for Tubocurarine (4) 10
NOE, RDC, and Structure Data for Vincristine (5) 12
Atomic Coordinates for Compounds (1)-(5) 14

## Formulas and Atom Numbering of Compounds (1)-(5)

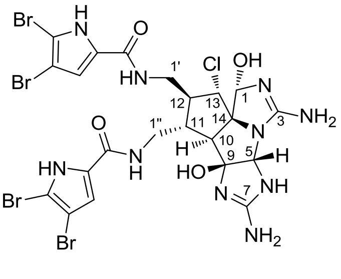

Axinellamine A (**1**)

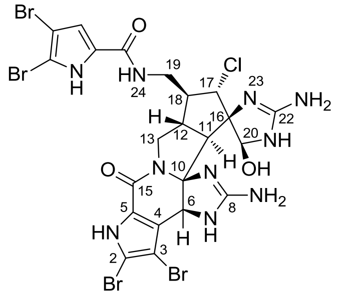

Tetrabromostyloguanidine (**2**)

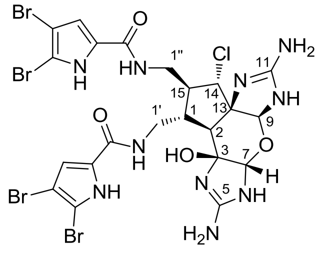

3,7-*epi*-Massadine chloride (**3**)

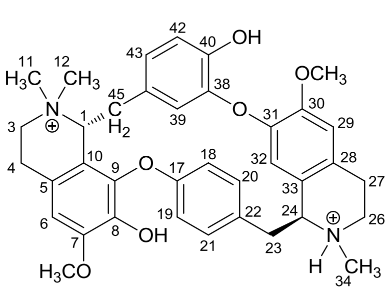

Tubocurarine (**4**)

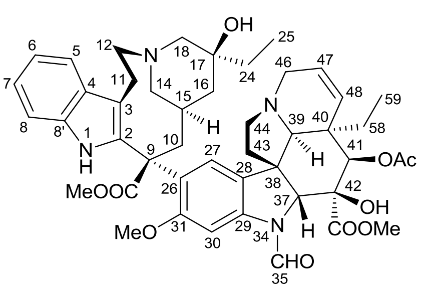

Vincristine (**5**)

# NOE and Structure Data for Axinellamine A (1)

Table S1. NOE data used for Axinellamine (**1**) ($N = 37$ NOEs, the NMR-derived expectation values are denoted as $d_{mean}$, and the allowed lower and upper bonds – usually $d_{mean} \pm 10\%$ – are labeled as $d_{min} \cdots d_{max}$; the averaged distances back-calculated from structural data are labelled $d_{calc}$, and the corresponding residuals are listed only if this value falls out of range, i.e. $d_{calc} < d_{min}$ or $d_{calc} > d_{max}$; all distance are given in [Å]). The NOE labels refer to the formula shown on the right, where given as e.g. $H^A|H^B$, NOEs were recalculated from structures as $r^{-6}$ averages over all pairs of atom-atom distances.

| **NOE** | | **distances [Å]** | | | |
|---|---|---|---|---|---|
| | | $d_{mean}$ | $d_{min} \cdots d_{max}$ | $d_{calc}$ | $\Delta d$ |
| H1 | H10 | 3.319 | 2.987-3.651 | 3.662 | 0.011 |
| H1 | H12 | 2.499 | 2.249-2.749 | 2.617 | - |
| H2 | H1 | 2.655 | 2.390-2.921 | 2.475 | - |
| H2 | H1X | 3.577 | 3.219-3.935 | 3.673 | - |
| H5 | H11 | 3.859 | 3.473-4.245 | 4.314 | 0.069 |
| H5 | H13 | 2.768 | 2.491-3.044 | 2.472 | -0.019 |
| H5 | H31 | 3.795 | 3.415-4.174 | 4.210 | 0.036 |
| H6 | H5 | 2.609 | 2.348-2.870 | 2.504 | - |
| H6 | H71 | 3.091 | 2.782-3.400 | 2.741 | -0.041 |
| H8 | H10 | 2.837 | 2.554-3.121 | 2.651 | - |
| H8 | H11 | 4.014 | 3.612-4.415 | 3.681 | - |
| H8 | H71 | 3.300 | 2.970-3.630 | 3.644 | 0.014 |
| H8 | H9X | 3.494 | 3.144-3.843 | 3.215 | - |
| H10 | H12 | 3.562 | 3.206-3.919 | 3.682 | - |
| H10 | H5 | 3.651 | 3.286-4.016 | 3.844 | - |
| H13 | H10 | 3.526 | 3.173-3.878 | 3.738 | - |
| H13 | H11 | 2.678 | 2.410-2.945 | 3.089 | 0.144 |
| H13 | H1A1 | 2.593 | 2.334-2.852 | 2.934 | 0.082 |
| H1X | H1 | 2.316 | 2.084-2.547 | 2.388 | - |
| H1X | H10 | 2.567 | 2.310-2.824 | 2.332 | - |
| H9X | H10 | 4.149 | 3.734-4.564 | 3.888 | - |
| H9X | H11 | 3.083 | 2.775-3.391 | 3.402 | 0.011 |
| H9X | H13 | 3.373 | 3.036-3.711 | 3.048 | - |
| H9X | H5 | 2.432 | 2.189-2.675 | 2.208 | - |
| H32 | H5 | 3.364 | 3.028-3.700 | 2.995 | -0.033 |
| H1A1 | H12 | 2.825 | 2.542-3.107 | 2.597 | - |
| H1A2 | H11 | 2.648 | 2.383-2.913 | 2.760 | - |
| H1A2 | H12 | 2.534 | 2.280-2.787 | 2.369 | - |
| H1A2 | H1A1 | 1.780 | 1.602-1.958 | 1.850 | - |
| H2A | H11 | 3.042 | 2.737-3.346 | 3.096 | - |
| H2A | H12 | 3.660 | 3.294-4.026 | 4.002 | - |
| H2A | H13 | 2.716 | 2.445-2.988 | 2.438 | -0.007 |
| H2A | H1A1 | 2.625 | 2.362-2.887 | 2.429 | - |
| H2B | H10 | 3.148 | 2.833-3.463 | 3.798 | 0.335 |
| H2B | H11 | 2.770 | 2.493-3.047 | 2.261 | -0.232 |
| H2B | H12 | 3.328 | 2.995-3.660 | 3.791 | 0.131 |
| H2B | H1A2 | 2.804 | 2.524-3.085 | 3.189 | 0.104 |

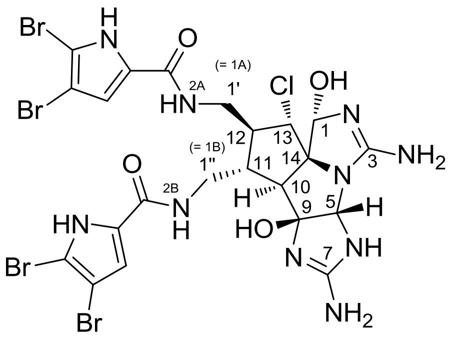

Axinellamine A (**1**)

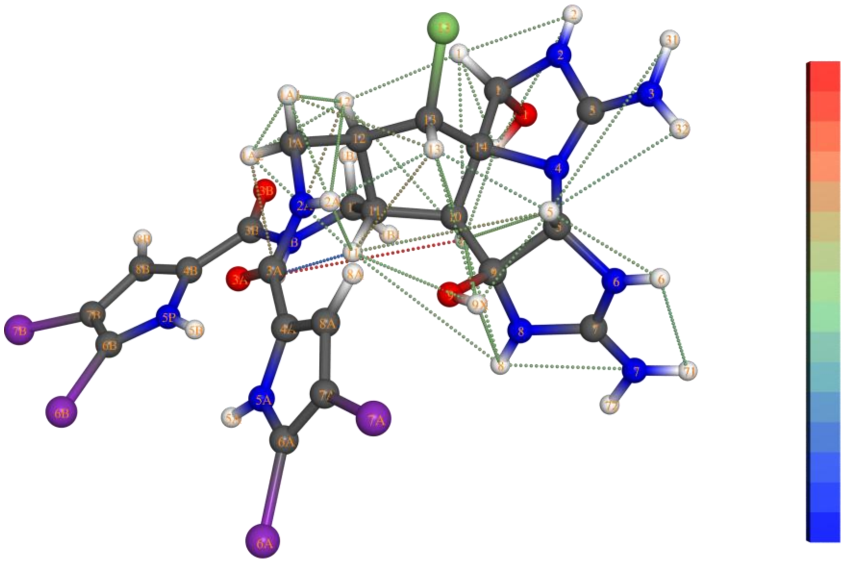

Figure S1. Plot of the best-fit (minimum pseudo energy) DG structure of Axinellamine A (**1**) with color-coded representation of all NOE contacts used in the configurational and conformational analysis. The color scale was adapted from calculated final NOE violations, ranging from -0.40 Å (blue) to +0.40 Å (red).

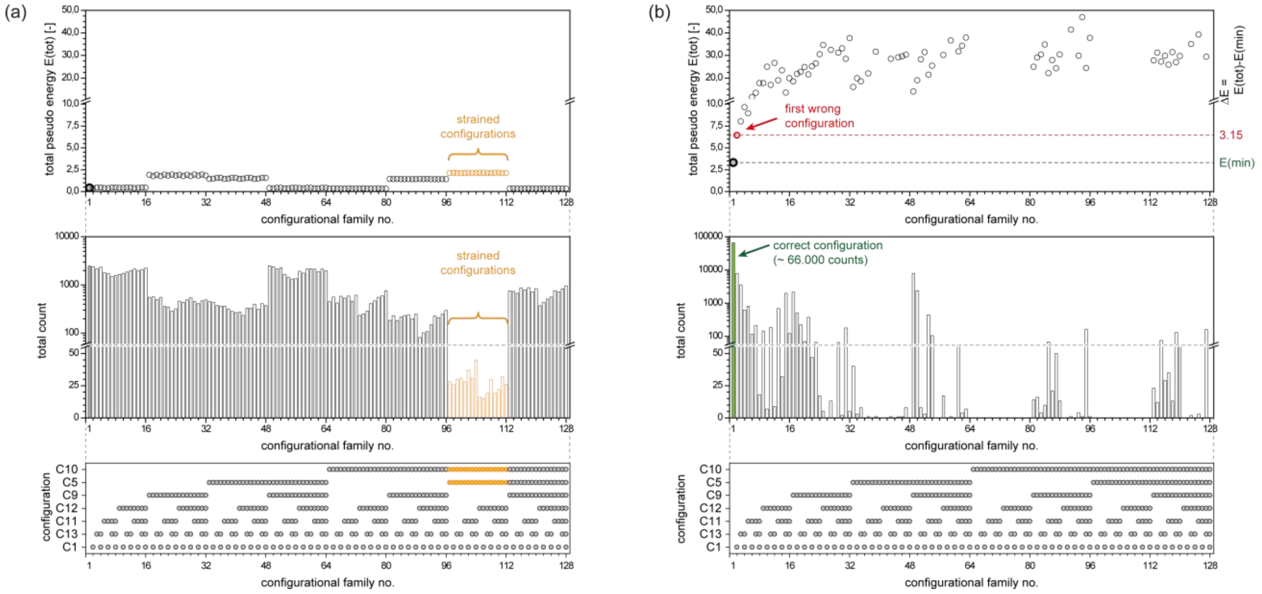


Figure S2.

(a) For Axinellamine A (**1**), an extended unrestricted floating-chirality DG calculation (100.000 structures) produces all 128 diastereomers (eight stereogenic centers, $2^{N-1}$ configurational families, only C14 was fixed in its configuration in order to avoid enantiomeric structures). In this simulation, only holonomic distance bounds (bond lengths) were used as restraints, and no NOE information was used to restrict configurational (and conformational) space. The *top plot* shows the minimum holonomic pseudo energy of each of the 128 diastereomers, and the *center plot* provides an overview on the total number (counts as columns) of structures generated in this simulation for each configuration. The *bottom plot* identifies each configuration generated: The rows of filled circles indicate for each configurational family which stereogenic center (C1, C13, C11, C12, C9, C5, and C10) is inverted in relation to the correct configuration of Axinellamine A (**1**, the correct configuration is labeled no. #1 on the left side of the plots), a missing circle indicates retention of stereogenic centers as compared to **1**. The DG approach samples even highly strained configurations (marked in orange, configurations no. #97-112), albeit these occur with significantly lower statistical sampling rates. In all these 16 highly strained diastereomers, at least C5 and C10 feature an inverted configuration, which leads to a two-fold *trans*-anellation of two of the five-membered rings (see Figure S3 below for a superposition plot of these structures).

(b) Using NOE data for Axinellamine (**1**) ($N = 37$ NOEs), an extended floating-chirality DG calculations (100.000 structures) produced 74 out of the 128 possible diastereomers, the *center* and *bottom plot* and visualize the individual configurational families and their relative counts as described in (a). The left most column displays the sampling rate for the correct configuration of Axinellamine A (**1**, about 66.000 correct structures were generated in this rDG simulation, note the logarithmic scale after the axis break), whereas the sampling rate of wrong configurations significantly drops as the total pseudo energy raises. The *top plot* shows the minimum total error (= pseudo energy) for each configurational family, the correct structure (left most entry labeled no. #1, large black circle) emerges as the global pseudo energy minimum structure ($E_{min} = 3.30$), the first wrong configuration (wrong configuration at C1, red circle) features a significantly higher pseudo energy ($\Delta E = 3.15$), and any alternate diastereomer is characterized by even higher pseudo energies (note the break in the scale of the axis). Nevertheless, DG efficiently samples all possible configurations under the restraints of the experimental NOE data, and the correct configuration of **1** emerges as the best-fit, lowest pseudo energy structure from all rDG simulations described here.

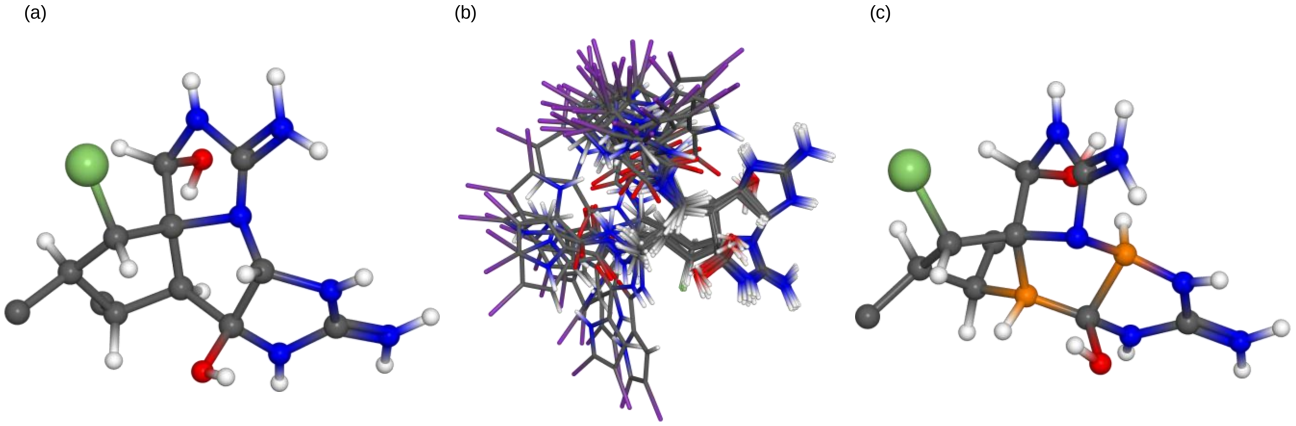


Figure S3. (a) Plot of central ring structure of Axinellamine A (**1**) with correct configuration of all stereogenic centers as identified from the rDG simulation described in Figure S2b; this structure is identical to the one shown in Figure S1. (b) Superposition of 16 highly strained diastereomers of **1**, identified from the unrestricted DG simulation as described in Figure S2a, and marked in these plots in orange. All these highly distorted structures feature a wrong configuration at C5 and C10 (atoms marked in orange in the plot on the right side (c)), and the structure shown in (c) corresponds to the configurational family labeled no. #97 in Figure S2b.

## NOE and Structure Data for Tetrabromostyloguanidine (2)

Table S2. NOE data used for Tetrabromostyloguanidine (**2**) ($N = 27$ NOEs, the NMR-derived expectation values are denoted as $d_{mean}$, and the allowed lower and upper bonds – usually $d_{mean} \pm 10\%$ – are labeled as $d_{min} \cdots d_{max}$; the averaged distances back-calculated from structural data are labelled $d_{calc}$, and the corresponding residuals are listed only if this value falls out of range, i.e. $d_{calc} < d_{min}$ or $d_{calc} > d_{max}$; all distance are given in [Å]). The NOE labels refer to the formula shown on the right, where given as e.g. $H^A|H^B$, NOEs were recalculated from structures as $r^{-6}$ averages over all pairs of atom-atom distances.

| NOE | | distances [Å] | | | |
|---|---|---|---|---|---|
| | | $d_{mean}$ | $d_{min} \cdots d_{max}$ | $d_{calc}$ | $\Delta d$ |
| H6 | H20X | 2.720 | 2.450-2.990 | 2.720 | - |
| H6 | H7 | 2.460 | 2.210-2.710 | 2.385 | - |
| H6 | H9 | 4.030 | 3.630-4.430 | 4.024 | - |
| H11 | H132 | 2.500 | 2.250-2.750 | 2.766 | 0.016 |
| H11 | H17 | 3.270 | 2.940-3.600 | 3.625 | 0.025 |
| H11 | H20X | 2.935 | 2.650-3.220 | 2.674 | - |
| H11 | H6 | 2.200 | 1.980-2.420 | 2.592 | 0.172 |
| H11 | H9 | 3.580 | 3.220-3.940 | 3.925 | - |
| H12 | H9 | 2.360 | 2.120-2.600 | 2.395 | - |
| H17 | H12 | 2.390 | 2.150-2.630 | 2.442 | - |
| H17 | H191 | 2.840 | 2.560-3.120 | 3.338 | 0.218 |
| H17 | H192 | 2.400 | 2.100-2.700 | 2.051 | -0.049 |
| H17 | H20 | 3.010 | 2.710-3.310 | 3.492 | 0.182 |
| H17 | H23 | 2.320 | 2.090-2.550 | 2.612 | 0.062 |
| H17 | H24 | 3.050 | 2.750-3.350 | 3.393 | 0.043 |
| H18 | H11 | 2.330 | 2.100-2.560 | 2.415 | - |
| H18 | H132 | 2.600 | 2.340-2.860 | 2.600 | - |
| H18 | H17 | 2.780 | 2.500-3.060 | 3.035 | - |
| H18 | H20 | 3.110 | 2.800-3.420 | 3.427 | 0.007 |
| H18 | H23 | 4.350 | 3.910-4.790 | 4.743 | - |
| H18 | H24 | 2.950 | 2.650-3.250 | 2.894 | - |
| H20 | H11 | 2.710 | 2.440-2.980 | 3.008 | 0.028 |
| H20 | H20X | 2.180 | 1.990-2.370 | 2.338 | - |
| H20 | H21 | 2.530 | 2.280-2.780 | 2.470 | - |
| H23 | H6 | 4.300 | 3.870-4.730 | 4.474 | - |
| H23 | H9 | 3.220 | 2.900-3.540 | 2.877 | -0.023 |
| H27 | H24 | 2.340 | 2.110-2.570 | 2.191 | - |

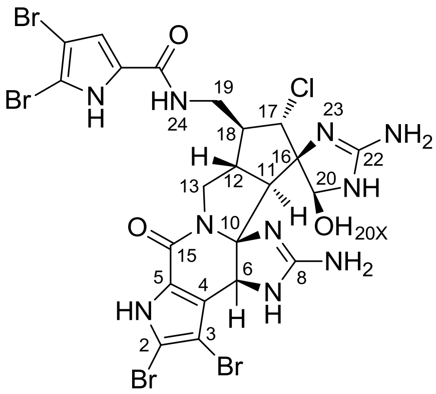

Tetrabromostyloguanidine (**2**)

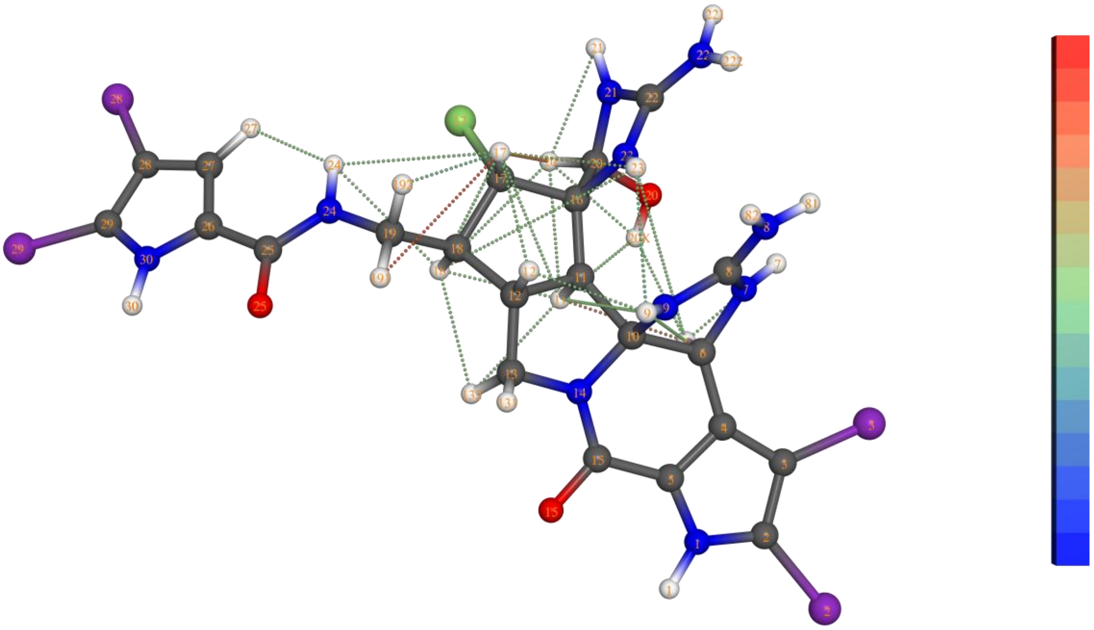

Figure S4. Plot of the best-fit (minimum pseudo energy) DG structure of Tetrabromostyloguanidine (**2**) with color-coded representation of all NOE contacts used in the configurational and conformational analysis. The color scale was adapted from calculated final NOE violations, ranging from -0.40 Å (blue) to +0.40 Å (red).

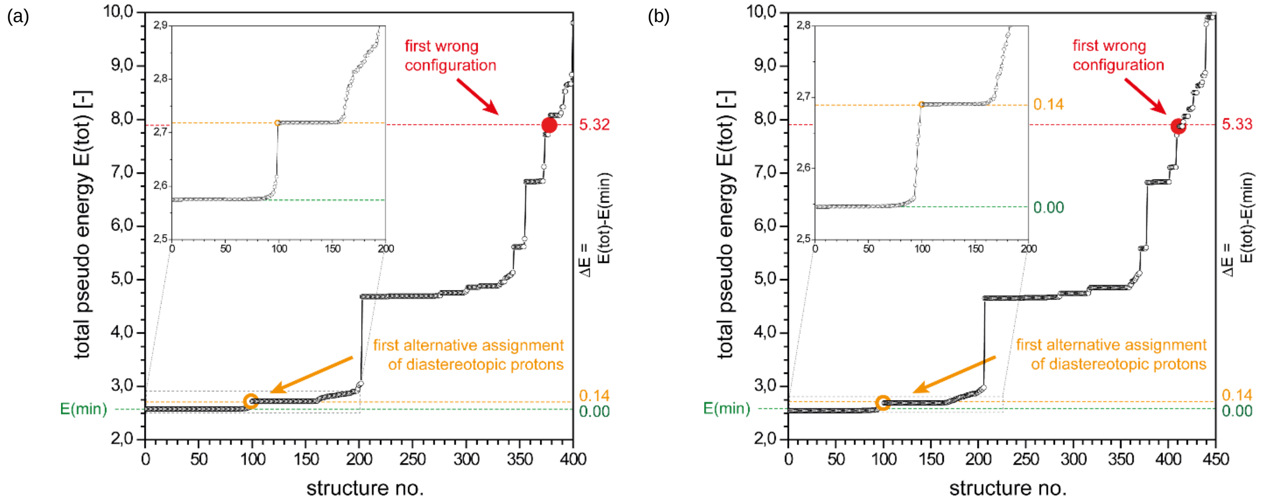


Figure S5.

(a) The *top left* plot shows pseudo energy sorted and ranked rDG structures of Tetrabromostyloguanidine (**2**) as discussed in the main paper. In this rDG simulation, a chiral volume restraint arbitrarily has been applied to a single stereogenic center (i.e. C10) in order to avoid enantiomeric structures.

(b) On the *top right*, the plot shows the corresponding pseudo energy of ranked rDG structures emerging from a simulation which did not use any chiral restraints at all, except on planar $sp^2$-type centers ($V_{chir} = 0$). The features of this plot, and in particular the energy steps are identical to the plot shown in (a), thus indicating that the arbitrary use of a single chiral volume restraint does not affect the final results of configurational and conformational analysis. In fact, this simulation produced the enantiomeric structures as shown below (Figure S6).

(c) On the *bottom right*, single chiral volume restraints were applied in rDG simulations on Tetrabromostyloguanidine (**2**), each using one of the eight stereogenic centers as a restrained and fixed reference. Restraining either one of C6, C10, C11, C12, C16, C17, C18, or C20 produces within very narrow margins of error the same energy step characteristics that have already been manifested in plots (a) and (b). In all cases, the first small step in energy results from an alternative assignment of diastereotopic methylene protons (see inset plot), and the first wrong relative configuration of one of the eight stereogenic centers (indicated by the bold-face large symbols) is always characterized by a significantly higher error in its total pseudo energy.

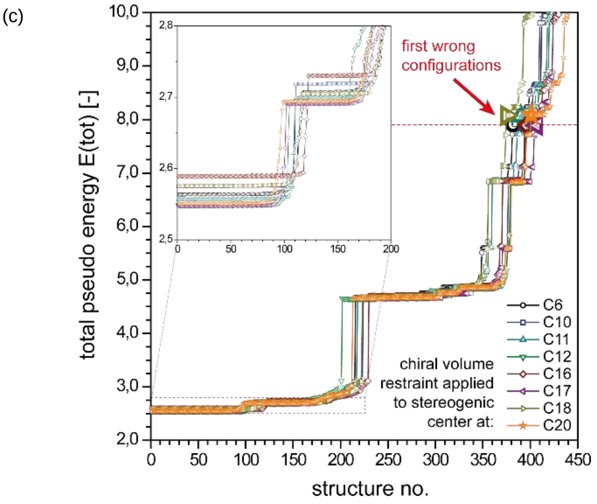


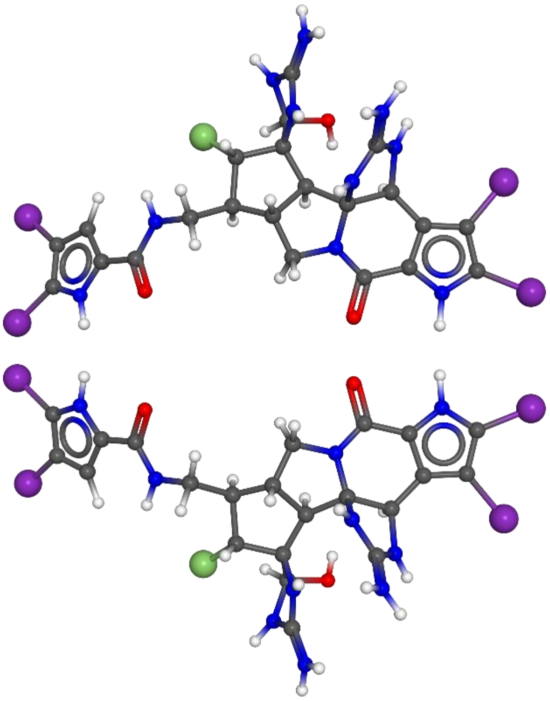

Figure S6. Ball-and-stick type representation of the first two lowest pseudo energy structures emerging from a rDG simulation on Tetrabromostyloguanidine (**2**) using no chiral restraints on any of the eight stereogenic centers, as shown in Figure S5b. These structures are actually exact enantiomers with identical pseudo energies.

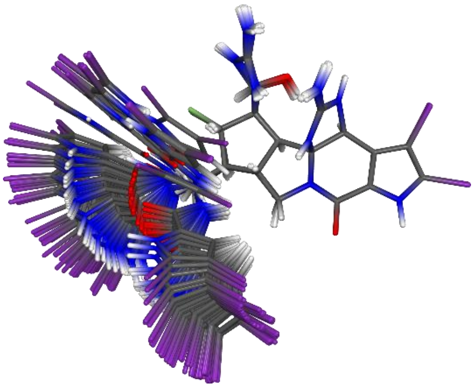

(a) chiral volume restraint applied to C6

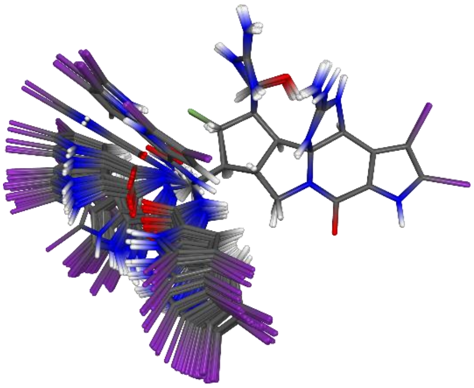

(b) chiral volume restraint applied to C10

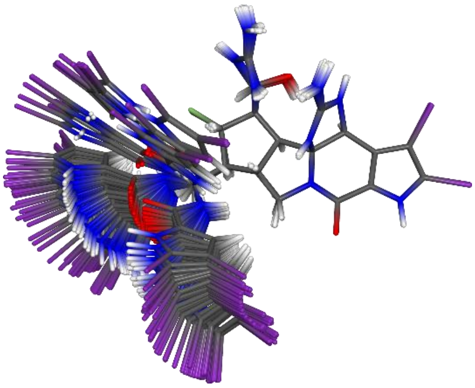

(c) chiral volume restraint applied to C11

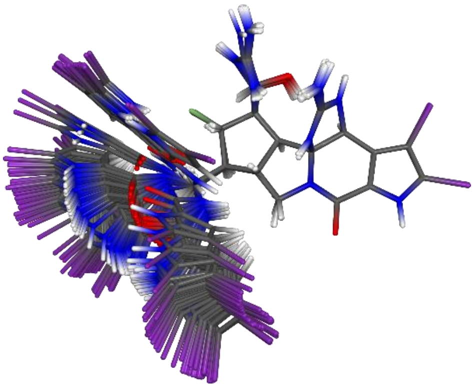

(d) chiral volume restraint applied to C12

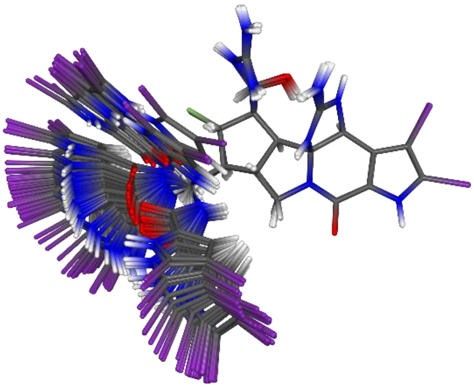

(e) chiral volume restraint applied to C16

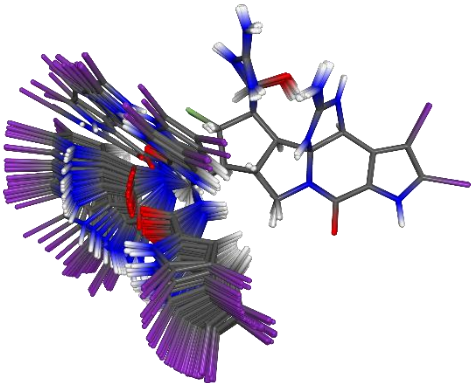

(f) chiral volume restraint applied to C17

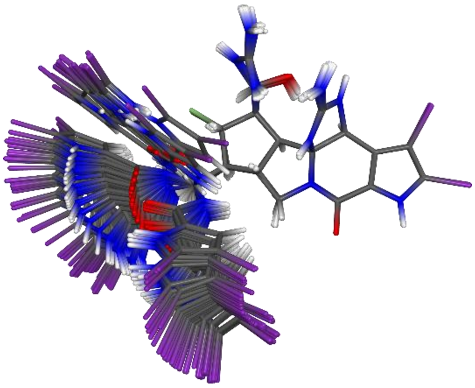

(g) chiral volume restraint applied to C18

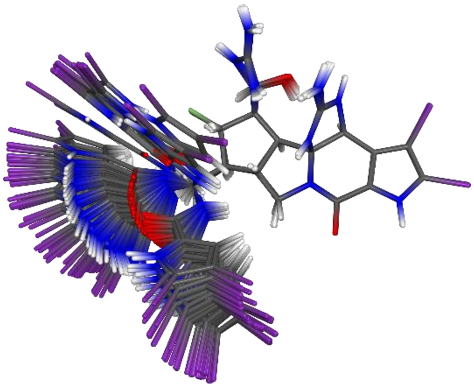

(h) chiral volume restraint applied to C20

Figure S7. Ball-and-stick type representations of all low pseudo energy structures up to the first wrong configuration emerging from rDG simulations on Tetrabromostyloguanidine (**2**) using a single chiral volume restraints on one of the eight stereogenic centers, as shown in Figure S5c. All structures shown here actually feature the same configuration of all eight stereogenic centers (a: 381, b: 393, c: 389, d: 395, e: 396, f: 406, g: 374, and h: 400 structures in total).

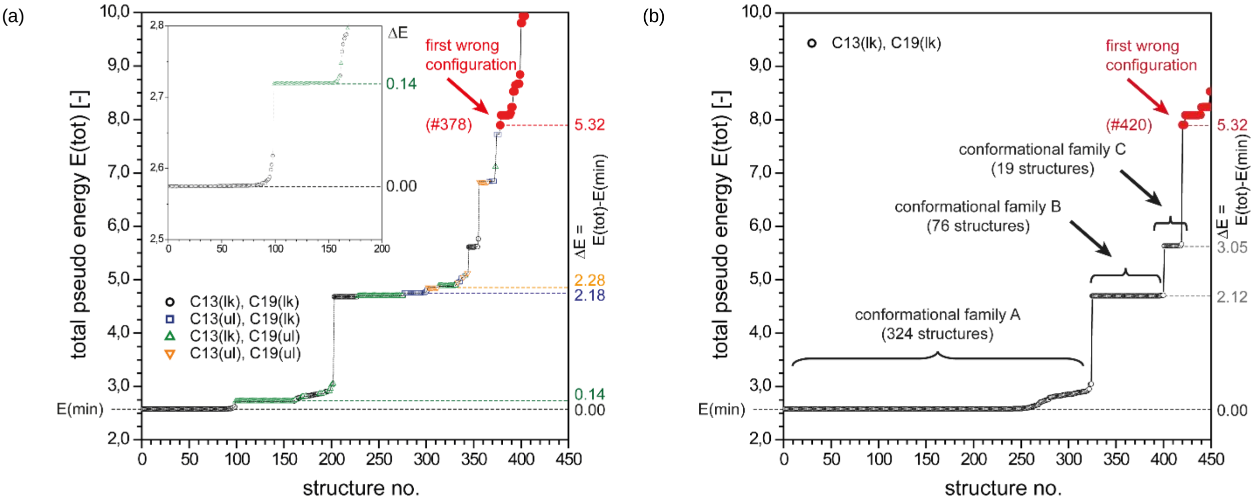


Figure S8. Detailed analysis of the assignments of diastereotopic methylene protons of Tetrabromostyloguanidine (**2**). (a) The *left plot* shows pseudo energy sorted and ranked rDG structures of **2** as discussed in the main paper. In addition, the color-coded symbols designate rankings of alternative assignments of CH2-protons (methylene groups C13 and C19), where the relative stereodescriptors "*lk*" (*like*) and "*ul*" (*unlike*) refer to the global pseudo energy minimum "*lk*/*lk*" assignment; the right scale gives $\Delta E$ values relative to $E(min)$. Different CH2-assignments were found (total number of structures generated: *lk*/*lk*: 162 (black), *ul*/*lk*: 44 (blue), *lk*/*ul*: 146 (green), and *ul*/*ul*: 25 (orange), with rankings of first occurrence *lk*/*lk*: #1 ($\Delta E = 0.00$), *ul*/*lk*: #277 ($\Delta E = 2.18$), *lk*/*ul*: #99 ($\Delta E = 0.14$), and *ul*/*ul*: #302 ($\Delta E = 2.28$)), which were ranked below the first wrong configuration of a stereogenic center (ranked no. #378, $\Delta E = 5.32$). (b) The *right plot* shows the pseudo energy of ranked rDG structures emerging from a simulation in which chiral volume restraints were used to restrict the pseudo configuration of both methylene groups in the correct low-energy configuration of plot (a). The sampling quality of correct *lk*/*lk* structures increases to 419 located below the occurrence of the first structure of wrong configuration ranked no. #420. The energy steps become significantly more pronounced as alternative assignments of methylene groups are discarded, and the low-energy structures cluster into three very distinct conformational families **A**-**C**. Below, superimposed structure plots of these different conformations are given (Figure S9).

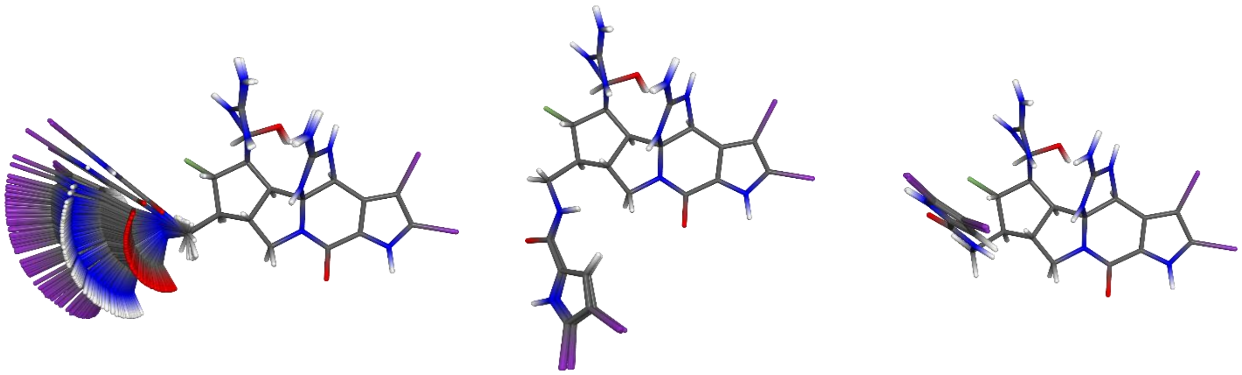

conformational family **A** (324 structures) $\Delta E = 0.00$ | conformational family **B** (76 structures) $\Delta E = 2.12$ | conformational family **C** (19 structures) $\Delta E = 3.05$

Figure S9. Superimposed structures for the conformational families **A**-**C** of Tetrabromostyloguanidine (**2**) as identified from the rDG simulation depicted in Figure S8b.

# NOE and Structure Data for 3,7-*epi*-Massadine chloride (3)

Table S3. NOE data used for 3,7-*epi*-Massadine chloride (**3**) ($N = 37$ NOEs, the NMR-derived expectation values are denoted as $d_{mean}$, and the allowed lower and upper bonds – usually $d_{mean} \pm 10\%$ – are labeled as $d_{min} \cdots d_{max}$; the averaged distances back-calculated from structural data are labelled $d_{calc}$, and the corresponding residuals are listed only if this value falls out of range, i.e. $d_{calc} < d_{min}$ or $d_{calc} > d_{max}$; all distance are given in [Å]). The NOE labels refer to the formula shown on the right, where given as e.g. $H^A|H^B$, NOEs were recalculated from structures as $r^{-6}$ averages over all pairs of atom-atom distances.

| NOE | | distances [Å] | | | |
|---|---|---|---|---|---|
| | | $d_{mean}$ | $d_{min} \cdots d_{max}$ | $d_{calc}$ | $\Delta d$ |
| H3X | H7 | 3.316 | 2.984-3.648 | 3.068 | - |
| H4 | H1 | 4.164 | 3.748-4.580 | 3.738 | -0.010 |
| H4 | H1A1 | 2.958 | 2.662-3.253 | 3.354 | 0.101 |
| H4 | H1A2 | 3.416 | 3.074-3.758 | 3.783 | 0.025 |
| H4 | H2 | 3.014 | 2.712-3.315 | 3.294 | - |
| H4 | H7 | 2.710 | 2.439-2.981 | 3.208 | 0.227 |
| H7 | H1 | 4.709 | 4.238-5.179 | 4.582 | - |
| H7 | H2 | 3.574 | 3.216-3.931 | 3.834 | - |
| H9 | H1 | 5.200 | 4.680-5.719 | 5.133 | - |
| H9 | H15 | 6.381 | 5.743-7.019 | 5.383 | -0.360 |
| H9 | H2 | 2.906 | 2.615-3.197 | 3.238 | 0.041 |
| H9 | H7 | 3.559 | 3.204-3.915 | 3.804 | - |
| H12 | H1 | 2.568 | 2.311-2.825 | 2.213 | -0.098 |
| H12 | H14 | 3.200 | 2.880-3.520 | 3.238 | - |
| H12 | H15 | 3.255 | 2.929-3.580 | 3.900 | 0.320 |
| H12 | H1B1 | 3.077 | 2.770-3.385 | 2.673 | -0.097 |
| H12 | H1B2 | 3.812 | 3.431-4.193 | 3.717 | - |
| H12 | H2 | 3.377 | 3.039-3.714 | 3.792 | 0.078 |
| H12 | H7 | 3.566 | 3.209-3.923 | 4.005 | 0.082 |
| H12 | H9 | 4.641 | 4.177-5.105 | 4.225 | - |
| H14 | H1 | 3.398 | 3.058-3.739 | 3.873 | 0.134 |
| H14 | H15 | 2.951 | 2.656-3.246 | 2.817 | - |
| H14 | H1B1 | 2.839 | 2.555-3.123 | 2.468 | -0.087 |
| H14 | H1B2 | 3.342 | 3.008-3.676 | 3.434 | - |
| H14 | H2 | 3.519 | 3.168-3.871 | 3.778 | - |
| H1A1 | H1 | 2.650 | 2.385-2.915 | 2.321 | -0.064 |
| H1A1 | H15 | 2.821 | 2.539-3.103 | 3.424 | 0.321 |
| H1A1 | H1A2 | 1.780 | 1.602-1.958 | 1.817 | - |
| H1A1 | H2 | 3.218 | 2.897-3.540 | 3.463 | - |
| H1A2 | H1 | 2.722 | 2.450-2.994 | 3.067 | 0.073 |
| H1A2 | H15 | 3.054 | 2.748-3.360 | 2.892 | - |
| H1A2 | H2 | 2.949 | 2.654-3.244 | 2.522 | -0.132 |
| H1B1 | H1 | 2.776 | 2.499-3.054 | 3.255 | 0.201 |
| H1B1 | H15 | 2.893 | 2.603-3.182 | 2.943 | - |
| H1B2 | H1 | 2.742 | 2.467-3.016 | 3.125 | 0.109 |
| H1B2 | H15 | 2.492 | 2.243-2.741 | 2.174 | -0.069 |
| H1B2 | H1B1 | 2.002 | 1.801-2.202 | 1.777 | -0.024 |

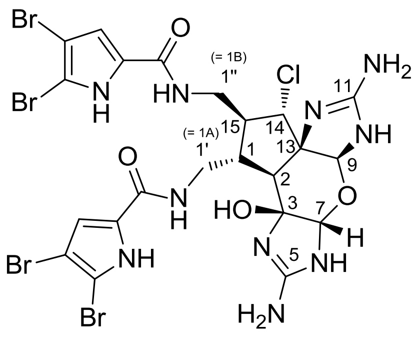


3,7-*epi*-Massadine chloride (**3**)

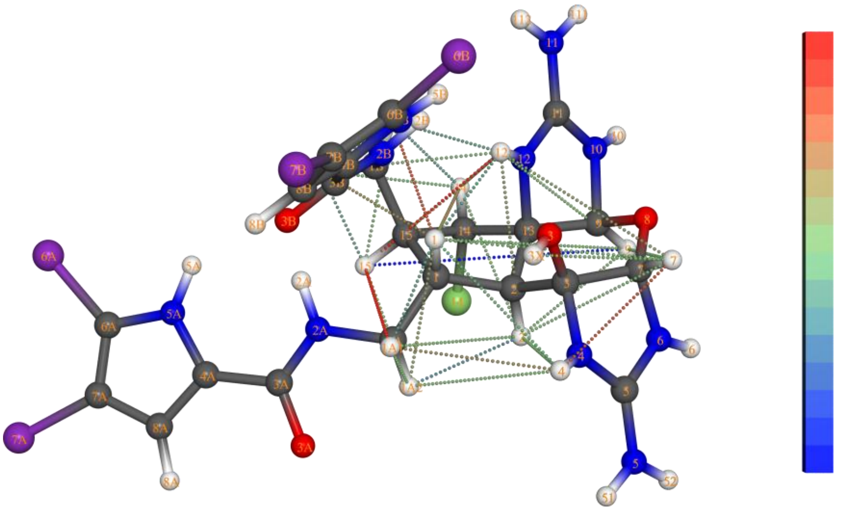

Figure S10. Plot of the best-fit (minimum pseudo energy) DG structure of 3,7-*epi*-Massadine chloride (**3**) with color-coded representation of all NOE contacts used in the configurational and conformational analysis. The color scale was adapted from calculated final NOE violations, ranging from -0.40 Å (blue) to +0.40 Å (red).

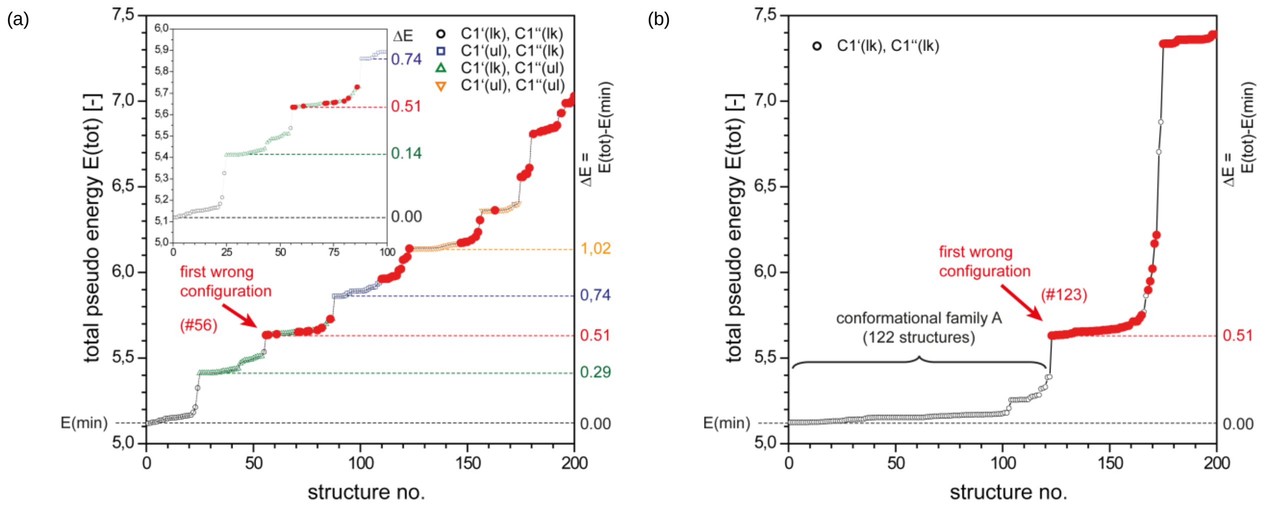


Figure S11. Detailed analysis of the assignments of diastereotopic methylene protons of 3,7-*epi*-Massadine chloride (**3**). (a) The *left plot* shows pseudo energy sorted and ranked rDG structures of **3** as discussed in the main paper. In addition, the color-coded symbols designate rankings of alternative assignments of CH2-protons (methylene groups C1' and C1''), where the relative stereodescriptors "*lk*" (*like*) and "*ul*" (*unlike*) refer to the global pseudo energy minimum "*lk*/*lk*" assignment; the right scale gives $\Delta E$ values relative to $E(min)$. Different CH2-assignments were found (total number of structures generated: *lk*/*lk*: 25 (black), *ul*/*lk*: 0 (blue), *lk*/*ul*: 30 (green), and *ul*/*ul*: 0 (orange), with rankings of first occurrence *lk*/*lk*: #1 ($\Delta E = 0.00$), *ul*/*lk*: #88 ($\Delta E = 0.74$), *lk*/*ul*: #25 ($\Delta E = 0.29$), and *ul*/*ul*: #124 ($\Delta E = 1.02$)), which were ranked below the first wrong configuration of a stereogenic center (ranked no. #56, $\Delta E = 0.51$). (b) The *right plot* shows the pseudo energy of ranked rDG structures emerging from a simulation in which chiral volume restraints were used to restrict the pseudo configuration of both methylene groups in the correct low-energy configuration of plot (a). The sampling quality of correct *lk*/*lk* structures increases to 122 located below the occurrence of the first structure of wrong configuration ranked no. #123. The energy steps become significantly more pronounced as alternative assignments of methylene groups are discarded, and the low-energy structures cluster into a single conformational family **A**. Below, a plot of these superimposed structures is given (Figure S12).

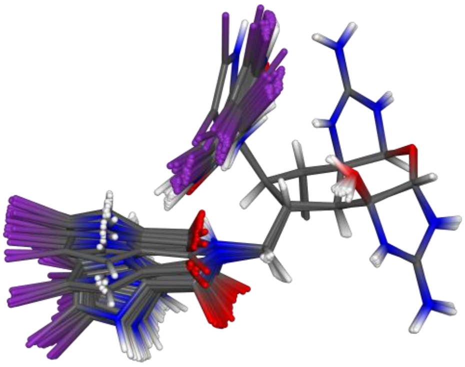

conformational family **A** (122 structures)
$\Delta E = 0.00$

Figure S12. Superimposed structures for the main low-pseudo energy conformational family **A** of 3,7-*epi*-Massadine chloride (**3**) as identified from the rDG simulation depicted in Figure S11b.

## NOE, RDC, and Structure Data for Tubocurarine (4)

The following table provides data on the RDCs ($D^{exp}$) used for Tubocurarine (**4**, three alignment media labeled [A]-[C]), their back-calculated values ($D^{calc}$), and residuals ($\Delta D = D^{exp} - D^{calc}$).

Table S4. RDC data used for Tubocurarine (**4**) ($N = 16$ RDCs, best-fit back-calculated RDCs $D^{calc}$ for each alignment medium (AM) labeled [A], [B], and [C], and residuals $\Delta D = D^{exp} - D^{calc}$, all values in [Hz]). Only bold face entries were used as parameters for the RDC analysis, regular font entries designate the calculated split terms of compound RDCs (where applicable, sums of RDCs are denoted as C-H[A]+C-H[B] $= D(CH^A) + D(CH^B)$, averages of RDCs are denoted as C-H[A]|C-H[B] $= (D(CH^A) + D(CH^B))/2$, methyl-RDCs were considered as three-state averages C-H[A]|H[B]|H[C]); the RDC labels refer to the formula shown on the right).

| RDC | AM [A] | | | AM [B] | | | AM [C] | | |
|---|---|---|---|---|---|---|---|---|---|
| | $D^{exp}$ | $D^{calc}$ | $\Delta D$ | $D^{exp}$ | $D^{calc}$ | $\Delta D$ | $D^{exp}$ | $D^{calc}$ | $\Delta D$ |
| **C1-H1** | **-9.13** | **-9.17** | **0.04** | **-4.33** | **-4.37** | **0.04** | **-0.04** | **-0.12** | **0.08** |
| **C3-H3A+C3-H3B** | **2.18** | **2.16** | **0.02** | **-11.53** | **-11.51** | **-0.02** | **24.69** | **24.68** | **0.01** |
| C3-H3A | - | -23.29 | - | - | -18.27 | - | - | 19.61 | - |
| C3-H3B | - | 25.45 | - | - | 6.76 | - | - | 5.07 | - |
| **C4-H4A+C4-H4B** | **19.99** | **19.98** | **0.01** | **6.87** | **6.89** | **-0.02** | **1.42** | **1.43** | **-0.01** |
| C4-H4A | - | 14.53 | - | - | 7.09 | - | - | 14.14 | - |
| C4-H4B | - | 5.45 | - | - | -0.20 | - | - | -12.71 | - |
| **C6-H6** | **-19.54** | **-19.57** | **0.03** | **8.00** | **8.04** | **-0.04** | **9.06** | **9.06** | **0.00** |
| **C18-H18\|C19-H19** | **-1.09** | **-1.15** | **0.06** | **-3.48** | **-3.50** | **0.02** | **4.76** | **4.68** | **0.08** |
| C18-H18 | - | 23.98 | - | - | 0.51 | - | - | -6.11 | - |
| C19-H19 | - | -26.28 | - | - | -7.50 | - | - | 15.47 | - |
| **C20-H20\|C21-H21** | **-1.55** | **-1.53** | **-0.02** | **-4.52** | **-4.37** | **-0.15** | **3.19** | **3.31** | **-0.12** |
| C20-H20 | - | -25.58 | - | - | -8.69 | - | - | 15.22 | - |
| C21-H21 | - | 22.52 | - | - | -0.04 | - | - | -8.60 | - |
| **C23-H23A+C23-H23B** | **-4.67** | **-4.66** | **-0.01** | **-16.38** | **-16.40** | **0.02** | **-5.03** | **-5.05** | **0.02** |
| C23-H23A | - | -23.42 | - | - | -18.64 | - | - | 19.00 | - |
| C23-H23B | - | 18.77 | - | - | 2.24 | - | - | -24.04 | - |
| **C24-H24** | **-24.11** | **-24.09** | **-0.02** | **-18.45** | **-18.59** | **0.14** | **19.33** | **19.33** | **0.00** |
| **C26-H26A+C26-H26B** | **7.52** | **7.48** | **0.04** | **10.11** | **10.16** | **-0.05** | **12.14** | **12.10** | **0.04** |
| C26-H26A | - | -6.30 | - | - | 0.75 | - | - | 24.81 | - |
| C26-H26B | - | 13.79 | - | - | 9.41 | - | - | -12.71 | - |
| **C27-H27A+C27-H27B** | **1.62** | **1.66** | **-0.04** | **3.54** | **3.61** | **-0.07** | **2.29** | **2.35** | **-0.06** |
| C27-H27A | - | -13.50 | - | - | -15.79 | - | - | 13.38 | - |
| C27-H27B | - | 15.16 | - | - | 19.40 | - | - | -11.03 | - |
| **C29-H29** | **-23.06** | **-22.88** | **-0.18** | **-2.94** | **-2.83** | **-0.11** | **10.20** | **10.37** | **-0.17** |
| **C32-H32** | **-22.20** | **-22.48** | **0.28** | **-3.65** | **-3.63** | **-0.02** | **10.36** | **10.15** | **0.21** |
| **C39-H39** | **12.20** | **12.33** | **-0.13** | **2.24** | **2.04** | **0.20** | **-3.98** | **-4.11** | **0.13** |
| **C42-H42** | **11.53** | **11.31** | **0.22** | **0.62** | **0.84** | **-0.22** | **-3.70** | **-3.63** | **-0.07** |
| **C43-H43** | **-24.30** | **-24.19** | **-0.11** | **-14.21** | **-14.32** | **0.11** | **17.12** | **17.14** | **-0.02** |
| **C45-H45A+C45-H45B** | **-22.14** | **-22.21** | **0.07** | **-26.90** | **-26.74** | **-0.16** | **6.48** | **6.48** | **0.00** |
| C45-H45A | - | -11.01 | - | - | -15.26 | - | - | -2.03 | - |
| C45-H45B | - | -11.20 | - | - | -11.47 | - | - | 8.51 | - |

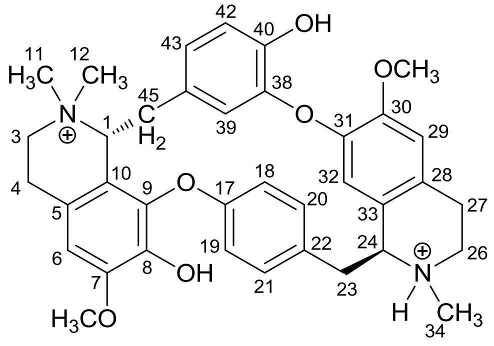


Tubocurarine (**4**)

Figure S13. Plot of the experimental RDCs ($D^{exp}$) vs. their back-calculated values ($D^{calc}$) used for Tubocurarine (**4**, three alignment media labeled [A]-[C]). The corresponding best-fit (lowest total *pseudo energy*) structure model (see plot below) was obtained from the DG simulation using all three AM data sets.

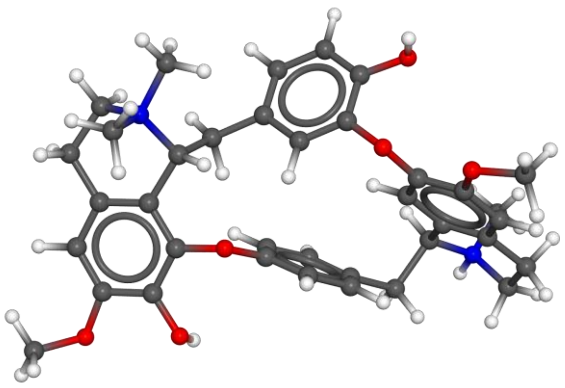

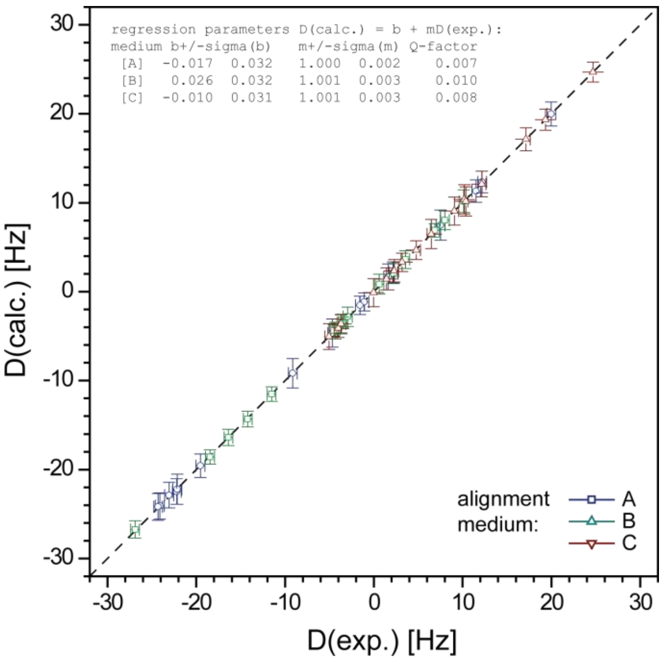

Table S5. NOE data used for Tubocurarine (**4**) ($N = 17$ NOEs, the NMR-derived expectation values are denoted as $d_{mean}$, and the allowed lower and upper bonds – usually $d_{mean} \pm 10\%$ – are labeled as $d_{min} \cdots d_{max}$; the averaged distances back-calculated from structural data are labelled $d_{calc}$, and the corresponding residuals are listed only if this value falls out of range, i.e. $d_{calc} < d_{min}$ or $d_{calc} > d_{max}$; all distance are given in [Å]). The NOE labels refer to the formula shown on the right, where given as e.g. $H^A|H^B$, NOEs were recalculated from structures as $r^{-6}$ averages over all pairs of atom-atom distances.

| NOE | | distances [Å] | | | |
|---|---|---|---|---|---|
| | | $d_{mean}$ | $d_{min} \cdots d_{max}$ | $d_{calc}$ | $\Delta d$ |
| H1 | H39 | 2.166 | 1.949-2.383 | 2.300 | - |
| H1 | H45A\|H45B | 2.639 | 2.375-2.903 | 2.654 | - |
| H3A\|H3B | H4A\|H4B | 2.473 | 2.226-2.720 | 2.488 | - |
| H4A\|H4B | H6 | 2.690 | 2.421-2.959 | 2.724 | - |
| H6 | H14A\|H14B\|H14C | 2.476 | 2.228-2.724 | 2.414 | - |
| H18\|H19 | H39 | 3.414 | 3.073-3.755 | 3.131 | - |
| H18\|H19 | H45A\|H45B | 2.525 | 2.272-2.778 | 2.548 | - |
| H20\|H21 | H23A\|H23B | 2.646 | 2.381-2.911 | 2.767 | - |
| H20\|H21 | H24 | 3.279 | 2.951-3.607 | 3.289 | - |
| H23A\|H23B | H24 | 3.024 | 2.722-3.326 | 2.663 | -0.059 |
| H23A\|H23B | H26A\|H26B | 2.336 | 2.102-2.570 | 2.561 | - |
| H24 | H32 | 2.338 | 2.104-2.572 | 2.385 | - |
| H26A\|H26B | H27A\|H27B | 2.438 | 2.194-2.682 | 2.480 | - |
| H27A\|H27B | H29 | 2.669 | 2.402-2.936 | 2.701 | - |
| H29 | H36A\|H36B\|H36C | 2.365 | 2.129-2.602 | 2.347 | - |
| H39 | H45A\|H45B | 2.972 | 2.675-3.269 | 2.879 | - |
| H43 | H45A\|H45B | 3.287 | 2.958-3.616 | 2.730 | -0.228 |

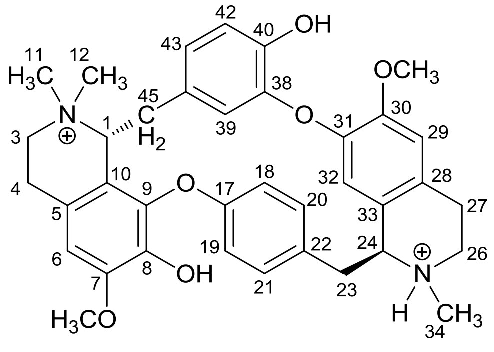


Tubocurarine (**4**)

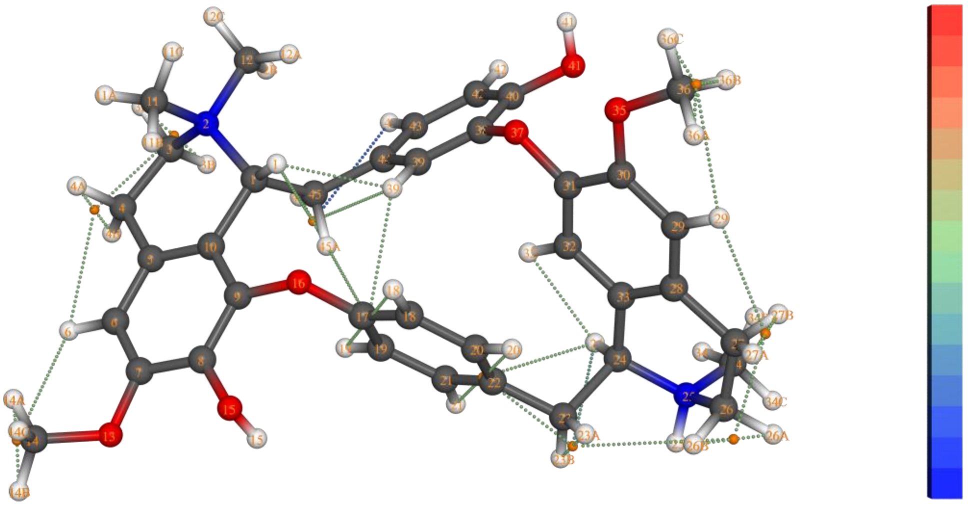

Figure S14. Plot of the best-fit (minimum pseudo energy) DG structure of Tubocurarine (**4**) with color-coded representation of all NOE contacts used in the configurational and conformational analysis. The color scale was adapted from calculated final NOE violations, ranging from -0.40 Å (blue) to +0.40 Å (red).

# NOE, RDC, and Structure Data for Vincristine (5)

The following table provides data on the RDCs ($D^{exp}$) used for Vincristine (**5**, three alignment media labeled [A]-[C]), their back-calculated values ($D^{calc}$), and residuals ($\Delta D = D^{exp} - D^{calc}$).

Table S6. RDC data used for Vincristine (**5**) ($N = 24$ RDCs, best-fit back-calculated RDCs $D^{calc}$ for each alignment medium (AM) labeled [A], [B], and [C], and residuals $\Delta D = D^{exp} - D^{calc}$, all values in [Hz]). Only bold face entries were used as parameters for the RDC analysis, regular font entries designate the calculated split terms of compound RDCs (where applicable, sums of RDCs are denoted as C-H$^A$+C-H$^B$ = $D(CH^A) + D(CH^B)$, averages of RDCs are denoted as C-H$^A$|C-H$^B$ = $(D(CH^A) + D(CH^B))/2$, methyl-RDCs were considered as three-state averages C-H$^A$|H$^B$|H$^C$); the RDC labels refer to the formula shown on the right).

| RDC | AM [A] | | | AM [B] | | | AM [C] | | |
|---|---|---|---|---|---|---|---|---|---|
| | $D^{exp}$ | $D^{calc}$ | $\Delta D$ | $D^{exp}$ | $D^{calc}$ | $\Delta D$ | $D^{exp}$ | $D^{calc}$ | $\Delta D$ |
| **C5-H5** | **18.60** | **18.68** | **-0.08** | **-25.15** | **-25.28** | **0.13** | **4.92** | **5.16** | **-0.24** |
| **C6-H6** | **4.47** | **4.49** | **-0.02** | **20.86** | **20.87** | **-0.01** | **19.24** | **19.33** | **-0.09** |
| **C7-H7** | **-0.13** | **-0.14** | **0.01** | **10.27** | **10.24** | **0.03** | **12.44** | **12.50** | **-0.06** |
| **C8-H8** | **19.98** | **19.92** | **0.06** | **-24.19** | **-24.18** | **-0.01** | **5.26** | **5.19** | **0.07** |
| **C10-H10A+C10-H10B** | **-4.79** | **-4.80** | **0.01** | **0.49** | **0.49** | **0.00** | **15.57** | **15.52** | **0.05** |
| C10-H10A | - | -16.23 | - | - | -22.31 | - | - | -0.54 | - |
| C10-H10B | - | 11.43 | - | - | 22.79 | - | - | 16.07 | - |
| **C11-H11A+C11-H11B** | **-8.62** | **-8.65** | **0.03** | **8.17** | **8.20** | **-0.03** | **-18.08** | **-18.08** | **0.00** |
| C11-H11A | - | -27.51 | - | - | -6.36 | - | - | -18.44 | - |
| C11-H11B | - | 18.85 | - | - | 14.56 | - | - | 0.36 | - |
| **C12-H12A+C12-H12B** | **29.22** | **29.20** | **0.02** | **-10.72** | **-10.65** | **-0.07** | **5.25** | **5.19** | **0.06** |
| C12-H12A | - | 16.79 | - | - | 13.76 | - | - | -1.84 | - |
| C12-H12B | - | 12.41 | - | - | -24.41 | - | - | 7.03 | - |
| **C14-H14A+C14-H14B** | **-13.51** | **-13.51** | **0.00** | **10.35** | **10.35** | **0.00** | **-11.89** | **-11.88** | **-0.01** |
| C14-H14A | - | -14.07 | - | - | 1.75 | - | - | -22.71 | - |
| C14-H14B | - | 0.56 | - | - | 8.60 | - | - | 10.83 | - |
| **C15-H15** | **-8.57** | **-8.57** | **0.00** | **-27.71** | **-27.68** | **-0.03** | **1.00** | **0.98** | **0.02** |
| **C16-H16A+C16-H16B** | **-3.06** | **-3.07** | **0.01** | **17.72** | **17.74** | **-0.02** | **-16.21** | **-16.22** | **0.01** |
| C16-H16A | - | 18.92 | - | - | 16.34 | - | - | 3.92 | - |
| C16-H16B | - | -21.99 | - | - | 1.40 | - | - | -20.14 | - |
| **C18-H18A+C18-H18B** | **-17.52** | **-17.52** | **0.00** | **-25.70** | **-25.70** | **0.00** | **-20.39** | **-20.38** | **-0.01** |
| C18-H18A | - | -12.45 | - | - | 0.78 | - | - | -23.66 | - |
| C18-H18B | - | -5.07 | - | - | -26.48 | - | - | 3.28 | - |
| **C24-H24A+C24-H24B** | **0.94** | **0.94** | **0.00** | **15.75** | **15.75** | **0.00** | **-9.08** | **-9.09** | **0.01** |
| C24-H24A | - | 13.46 | - | - | 20.27 | - | - | 15.55 | - |
| C24-H24B | - | -12.51 | - | - | -4.51 | - | - | -24.64 | - |
| **C27-H27** | **14.52** | **14.49** | **0.03** | **18.78** | **18.67** | **0.11** | **15.29** | **15.24** | **0.05** |
| **C30-H30** | **17.02** | **17.00** | **0.02** | **14.74** | **14.84** | **-0.10** | **14.06** | **14.14** | **-0.08** |
| **C35-H35** | **7.91** | **8.01** | **-0.10** | **6.56** | **6.36** | **0.20** | **-11.24** | **-11.17** | **-0.07** |
| **C37-H37** | **-16.61** | **-16.63** | **0.02** | **8.66** | **8.65** | **0.01** | **-15.02** | **-14.97** | **-0.05** |
| **C39-H39** | **-24.87** | **-24.93** | **0.06** | **2.00** | **1.90** | **0.10** | **4.18** | **4.31** | **-0.13** |
| **C41-H41** | **-5.03** | **-5.03** | **0.00** | **-4.67** | **-4.65** | **-0.02** | **-19.53** | **-19.49** | **-0.04** |
| **C43-H43A+C43-H43B** | **-30.83** | **-30.77** | **-0.06** | **3.45** | **3.46** | **-0.01** | **13.99** | **13.95** | **0.04** |
| C43-H43A | - | -16.03 | - | - | -24.48 | - | - | -1.86 | - |
| C43-H43B | - | -14.74 | - | - | 27.94 | - | - | 15.81 | - |
| **C44-H44A+C44-H44B** | **-14.42** | **-14.41** | **-0.01** | **6.81** | **6.79** | **0.02** | **2.47** | **2.45** | **0.02** |
| C44-H44A | - | 14.27 | - | - | 2.35 | - | - | 14.36 | - |
| C44-H44B | - | -28.68 | - | - | 4.44 | - | - | -11.91 | - |
| **C46-H46A+C46-H46B** | **-22.40** | **-22.40** | **0.00** | **-2.47** | **-2.48** | **0.01** | **-10.39** | **-10.39** | **0.00** |
| C46-H46A | - | -16.83 | - | - | -0.89 | - | - | -23.50 | - |
| C46-H46B | - | -5.57 | - | - | -1.59 | - | - | 13.11 | - |
| **C47-H47** | **-0.88** | **-0.95** | **0.07** | **-3.29** | **-3.27** | **-0.02** | **-0.91** | **-0.89** | **-0.02** |
| **C48-H48** | **15.49** | **15.47** | **0.02** | **-13.54** | **-13.56** | **0.02** | **3.53** | **3.48** | **0.05** |
| **C58-H58A+C58-H58B** | **22.26** | **22.26** | **0.00** | **-24.94** | **-24.94** | **0.00** | **-2.45** | **-2.44** | **-0.01** |
| C58-H58A | - | 25.15 | - | - | -16.08 | - | - | 1.17 | - |
| C58-H58B | - | -2.89 | - | - | -8.86 | - | - | -3.61 | - |

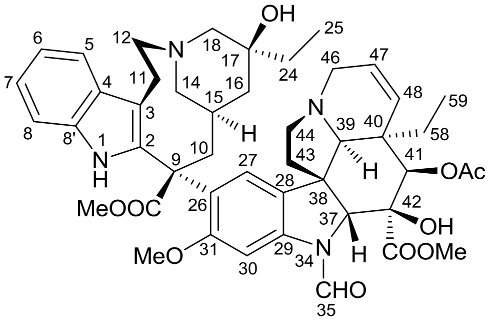


Vincristine (**5**)

Figure S15. Plot of the experimental RDCs ($D^{exp}$) vs. their back-calculated values ($D^{calc}$) used for Vincrsitine (**5**, three alignment media labeled [A]-[C]). The corresponding best-fit (lowest total *pseudo energy*) structure model (see plot below) was obtained from the DG simulation using all three AM data sets.

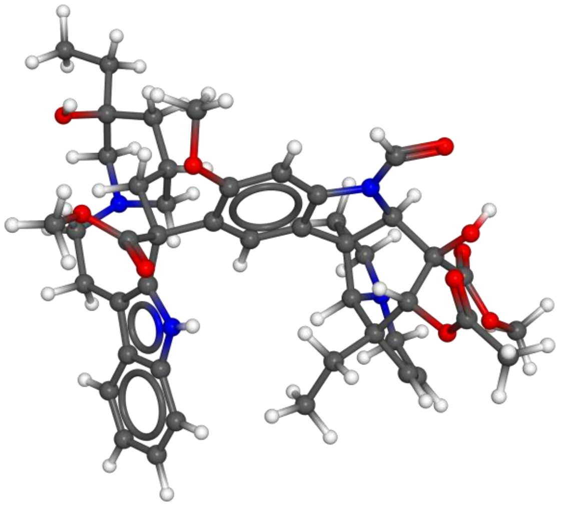

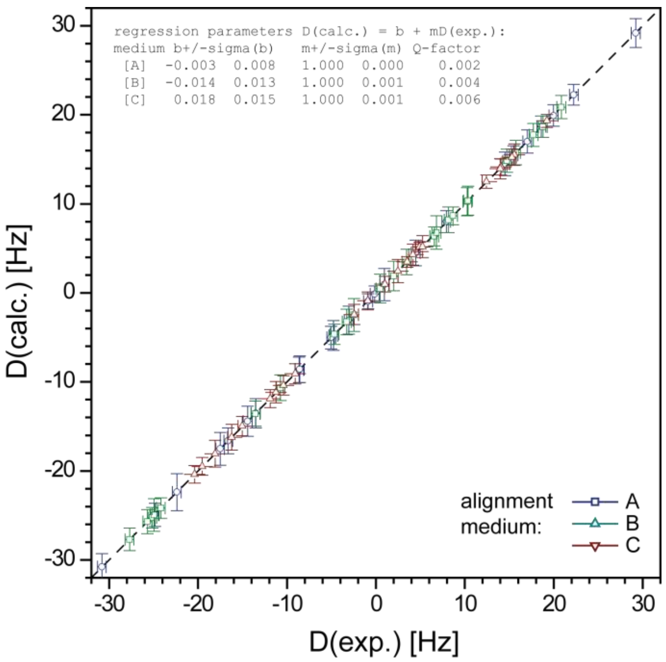


Table S7. NOE data used for Vincristine (**5**) ($N = 23$ NOEs, the NMR-derived expectation values are denoted as $d_{mean}$, and the allowed lower and upper bonds – usually $d_{mean} \pm 10\%$ – are labeled as $d_{min} \cdots d_{max}$; the averaged distances back-calculated from structural data are labelled $d_{calc}$, and the corresponding residuals are listed only if this value falls out of range, i.e. $d_{calc} < d_{min}$ or $d_{calc} > d_{max}$; all distance are given in [Å]). The NOE labels refer to the formula shown on the right, where given as e.g. $H^A|H^B$, NOEs were recalculated from structures as $r^{-6}$ averages over all pairs of atom-atom distances.

| NOE | | distances [Å] | | | |
|---|---|---|---|---|---|
| | | $d_{mean}$ | $d_{min} \cdots d_{max}$ | $d_{calc}$ | $\Delta d$ |
| H30 | H35 | 2.206 | 1.985-2.426 | 2.149 | - |
| H27 | H39 | 2.458 | 2.212-2.704 | 2.565 | - |
| H1 | H27 | 2.672 | 2.405-2.939 | 2.877 | - |
| H15 | H27 | 2.684 | 2.416-2.952 | 2.752 | - |
| H10A\|H10B | H12A\|H12B | 2.176 | 1.958-2.394 | 2.290 | - |
| H10A\|H10B | H16A\|H16B | 2.783 | 2.505-3.061 | 2.809 | - |
| H12A\|H12B | H18A\|H18B | 2.705 | 2.435-2.976 | 2.703 | - |
| H14A\|H14B | H16A\|H16B | 3.100 | 2.790-3.410 | 3.162 | - |
| H14A\|H14B | H18A\|H18B | 2.926 | 2.633-3.219 | 2.940 | - |
| H14A\|H14B | H27 | 2.598 | 2.338-2.858 | 2.621 | - |
| H16A\|H16B | H18A\|H18B | 3.292 | 2.963-3.621 | 3.310 | - |
| H27 | H43A\|H43B | 2.894 | 2.605-3.183 | 2.725 | - |
| H27 | H58A\|H58B | 3.304 | 2.974-3.634 | 3.563 | - |
| H37 | H43A\|H43B | 2.562 | 2.306-2.818 | 2.533 | - |
| H37 | H44A\|H44B | 3.287 | 2.958-3.616 | 3.319 | - |
| H39 | H41 | 3.463 | 3.117-3.809 | 3.448 | - |
| H39 | H43A\|H43B | 3.140 | 2.826-3.454 | 3.190 | - |
| H39 | H44A\|H44B | 3.330 | 2.997-3.663 | 3.415 | - |
| H39 | H46A\|H46B | 3.014 | 2.713-3.315 | 3.091 | - |
| H39 | H58A\|H58B | 2.653 | 2.388-2.918 | 2.611 | - |
| H41 | H58A\|H58B | 2.361 | 2.125-2.597 | 2.369 | - |
| H44A\|H44B | H46A\|H46B | 2.829 | 2.546-3.112 | 2.798 | - |
| H48 | H58A\|H58B | 2.864 | 2.578-3.150 | 2.946 | - |

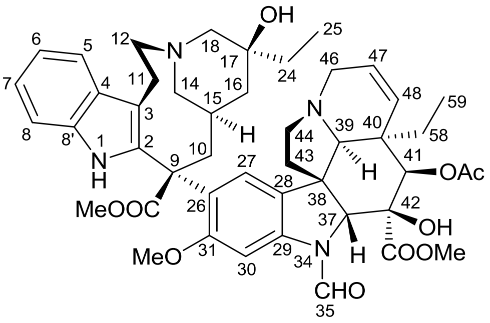

Vincristine (**5**)

Figure S16. Plot of the best-fit (minimum pseudo energy) DG structure of Vincrsitine (**5**) with color-coded representation of all NOE contacts used in the configurational and conformational analysis. The color scale was adapted from calculated final NOE violations, ranging from -0.40 Å (blue) to +0.40 Å (red).

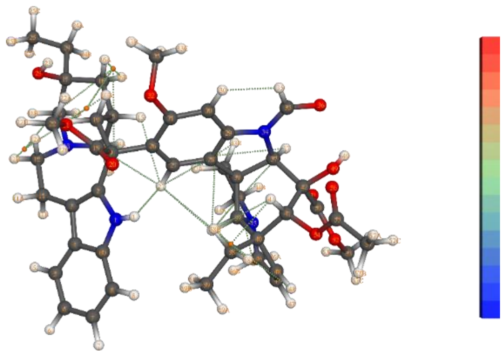

## Atomic Coordinates for Compounds (1)-(5)

The following tables provide the atomic coordinates for the final best-fit (minimum pseudo energy) DG structures of (**1**)-(**5**), copy-and-paste for further usage:

Table S8. rDG best-fit structures (atomic coordinates) for compounds (**1**)-(**5**).

| Axinellamine A (1) | Tetrabromostylo-guanidine (2) | 3,7-epi-Massadine chloride (3) | Tubocurarine (4) | Vincristine (5) |
|---|---|---|---|---|
| 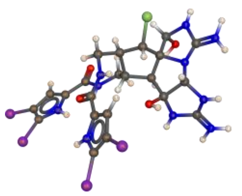 | 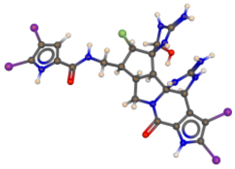 | 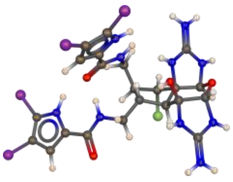 | 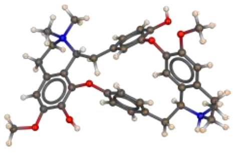 | 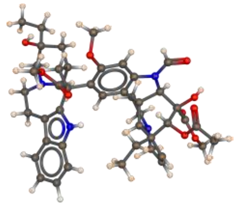 |

**Axinellamine A (1)**

| | | | |
|---|---|---|---|
| C1 | -2.881472 | -0.668349 | -2.430370 |
| O1 | -2.981222 | 0.523986 | -3.235802 |
| N2 | -4.324663 | -0.969276 | -2.138918 |
| C3 | -4.549924 | -0.249399 | -0.892619 |
| N3 | -5.768046 | -0.295439 | -0.449255 |
| N4 | -3.502670 | 0.325330 | -0.422748 |
| C5 | -3.196253 | 0.748263 | 0.886095 |
| N6 | -3.749854 | 2.034221 | 1.453231 |
| C7 | -2.885993 | 2.998609 | 1.166591 |
| N7 | -3.171249 | 4.301521 | 1.111965 |
| N8 | -1.555804 | 2.541736 | 0.892792 |
| C9 | -1.646724 | 1.136881 | 0.798655 |
| O9 | -0.939405 | 0.557697 | 1.882486 |
| C10 | -1.248805 | 0.450979 | -0.572135 |
| C11 | 0.116996 | -0.267510 | -0.813149 |
| C12 | -0.331439 | -1.821680 | -0.875671 |
| C13 | -1.817884 | -1.816823 | -0.377278 |
| C14 | -2.288783 | -0.464970 | -1.021748 |
| Cl13 | -2.831937 | -3.200003 | -0.884687 |
| H1 | -2.453310 | -1.437547 | -3.073108 |
| H1X | -2.120933 | 0.924197 | -3.188836 |
| H2 | -4.874481 | -1.424519 | -2.561413 |
| H5 | -3.338464 | -0.048025 | 1.616712 |
| H6 | -4.620894 | 2.074139 | 1.964952 |
| H8 | -0.852556 | 3.125308 | 0.811365 |
| H9X | -1.469501 | 0.547905 | 2.630721 |
| H10 | -1.071173 | 1.356981 | -1.151538 |
| H11 | 0.698458 | -0.136580 | 0.099463 |
| H12 | -0.217453 | -2.143055 | -1.910961 |
| H13 | -1.837498 | -1.791260 | 0.712246 |
| H31 | -6.512999 | -0.856523 | -1.027783 |
| H32 | -6.077495 | 0.173576 | 0.426819 |
| H71 | -4.111660 | 4.681821 | 1.293034 |
| H72 | -2.353895 | 4.969231 | 0.855326 |
| C1A | 0.606415 | -2.779441 | -0.020340 |
| N2A | 0.752941 | -2.217826 | 1.345091 |
| C3A | 1.821056 | -1.409536 | 1.679714 |
| O3A | 2.707677 | -1.089074 | 0.880762 |
| C4A | 1.804362 | -0.961023 | 3.098397 |
| N5A | 2.747961 | -0.145214 | 3.712314 |
| C6A | 2.416978 | 0.064389 | 5.029910 |
| C7A | 1.228437 | -0.635919 | 5.269095 |
| C8A | 0.835502 | -1.282860 | 4.063682 |
| Br6A | 3.433965 | 1.116688 | 6.227979 |
| Br7A | 0.249332 | -0.752861 | 6.890224 |
| H1A1 | 0.205893 | -3.790653 | 0.051292 |
| H1A2 | 1.590515 | -2.683714 | -0.479126 |
| H2A | 0.079800 | -2.443317 | 2.068857 |
| H5A | 3.597427 | 0.254302 | 3.230033 |
| H8A | -0.049253 | -1.905327 | 3.930105 |
| C1B | 0.769119 | 0.212176 | -2.160517 |
| N2B | 2.232033 | 0.246003 | -2.021631 |
| C3B | 3.079549 | 0.220478 | -3.091402 |
| O3B | 2.697079 | 0.167026 | -4.272778 |
| C4B | 4.519527 | 0.262689 | -2.711158 |
| N5B | 5.023270 | 0.322780 | -1.418525 |
| C6B | 6.398470 | 0.348101 | -1.440378 |
| C7B | 6.785626 | 0.302897 | -2.786256 |
| C8B | 5.614824 | 0.249234 | -3.589868 |
| Br6B | 7.483614 | 0.429131 | 0.105064 |
| Br7B | 8.546853 | 0.308894 | -3.492731 |
| H1B1 | 0.478137 | 1.240751 | -2.373739 |
| H1B2 | 0.515556 | -0.447754 | -2.990154 |
| H2B | 2.572969 | 0.288307 | -1.090752 |
| H5B | 4.432486 | 0.345702 | -0.544018 |
| H8B | 5.587314 | 0.206211 | -4.678671 |

**Tetrabromostylo-guanidine (2)**

| | | | |
|---|---|---|---|
| N1 | -5.497115 | -2.659226 | -0.082654 |
| C2 | -6.538583 | -1.858724 | -0.374497 |
| C3 | -6.048074 | -0.554340 | -0.726067 |
| C4 | -4.654755 | -0.637226 | -0.626045 |
| C5 | -4.355385 | -1.965678 | -0.220149 |
| C6 | -3.505014 | 0.282697 | -0.819907 |
| N7 | -3.381257 | 1.516311 | -1.613506 |
| C8 | -2.574322 | 1.118146 | -2.678949 |
| N8 | -2.433313 | 1.772453 | -3.799864 |
| N9 | -1.947657 | -0.089088 | -2.346162 |
| C10 | -2.097230 | -0.317940 | -0.895695 |
| C11 | -0.819644 | 0.309681 | -0.284862 |
| C12 | 0.219656 | -0.918148 | -0.599948 |
| C13 | -0.564104 | -2.116785 | -0.270724 |
| N14 | -1.938463 | -1.595039 | -0.197071 |
| C15 | -2.957430 | -2.417900 | -0.005800 |
| O15 | -2.765337 | -3.673532 | 0.367586 |
| C16 | 0.023830 | 1.548085 | -0.332211 |
| C17 | 1.476331 | 1.067454 | -0.122251 |
| C18 | 1.417536 | -0.459007 | 0.280407 |
| C19 | 2.781694 | -1.167903 | -0.114220 |
| C20 | -0.202076 | 2.835896 | 0.609680 |
| O20 | -1.586609 | 3.136011 | 0.915965 |
| N21 | 0.462156 | 3.871380 | -0.189410 |
| C22 | 0.073463 | 3.629009 | -1.518808 |
| N22 | -0.142030 | 4.550880 | -2.401389 |
| N23 | -0.031625 | 2.273682 | -1.654568 |
| N24 | 3.767756 | -1.070334 | 0.981465 |
| C25 | 4.268856 | -2.196097 | 1.596671 |
| O25 | 3.913764 | -3.334219 | 1.257292 |
| C26 | 5.276748 | -2.068179 | 2.713066 |
| C27 | 5.794256 | -0.848844 | 3.228235 |
| C28 | 6.691558 | -1.192090 | 4.244494 |
| C29 | 6.694675 | -2.623439 | 4.319526 |
| N30 | 5.839099 | -3.090318 | 3.390226 |
| Cl17 | 2.472443 | 2.026108 | 0.934810 |
| Br2 | -8.361897 | -2.358061 | -0.323050 |
| Br3 | -7.069210 | 0.959667 | -1.218473 |
| Br28 | 7.700941 | 0.003184 | 5.308072 |
| Br29 | 7.725115 | -3.664734 | 5.518372 |
| H1 | -5.551045 | -3.659027 | 0.211000 |
| H6 | -3.449835 | 0.716873 | 0.178364 |
| H7 | -3.771808 | 2.430457 | -1.449313 |
| H81 | -2.939299 | 2.640893 | -3.930821 |
| H82 | -1.829580 | 1.425517 | -4.533246 |
| H9 | -1.467994 | -0.697509 | -3.025454 |
| H11 | -0.994275 | 0.145341 | 0.778433 |
| H12 | 0.489514 | -0.801083 | -1.649506 |
| H131 | -0.566412 | -2.927831 | -0.998940 |
| H132 | -0.361982 | -2.546967 | 0.710189 |
| H17 | 1.975379 | 1.054659 | -1.091213 |
| H18 | 1.233260 | -0.594249 | 1.346170 |
| H191 | 2.597973 | -2.171165 | -0.498685 |
| H192 | 3.230208 | -0.556021 | -0.896866 |
| H20 | 0.373260 | 2.718050 | 1.527939 |
| H20X | -1.955348 | 2.510658 | 1.573326 |
| H21 | 1.071333 | 4.613241 | 0.105202 |
| H221 | -0.024822 | 5.536837 | -2.143301 |
| H222 | -0.424573 | 4.326457 | -3.342484 |
| H23 | -0.067073 | 1.770859 | -2.553648 |
| H24 | 4.087769 | -0.121649 | 1.289325 |
| H27 | 5.545472 | 0.160486 | 2.900425 |
| H30 | 5.638043 | -4.098942 | 3.216470 |

**3,7-epi-Massadine chloride (3)**

| | | | |
|---|---|---|---|
| C1 | -0.288321 | -0.446101 | 0.084231 |
| C2 | -1.819544 | -0.586497 | -0.197564 |
| C3 | -2.270217 | -0.024611 | -1.602974 |
| O3 | -1.566062 | 1.057332 | -2.087571 |
| N4 | -2.280952 | -1.156687 | -2.634298 |
| C5 | -3.447196 | -1.927778 | -2.424102 |
| N5 | -3.667261 | -3.259126 | -2.830780 |
| N6 | -4.374152 | -1.120815 | -1.723730 |
| C7 | -3.804305 | 0.262466 | -1.561674 |
| O8 | -4.317745 | 0.981280 | -0.529668 |
| C9 | -3.939253 | 0.487428 | 0.833629 |
| N10 | -4.087030 | 1.788590 | 1.716981 |
| C11 | -2.983745 | 2.582060 | 1.393628 |
| N11 | -2.825251 | 3.931376 | 1.743880 |
| N12 | -2.062277 | 1.823131 | 0.666185 |
| C13 | -2.473969 | 0.293914 | 0.901396 |
| C14 | -1.634136 | -0.086251 | 2.121581 |
| C15 | -0.172525 | -0.045106 | 1.598072 |
| Cl14 | -1.942049 | -1.760608 | 2.717294 |
| H1 | 0.062163 | 0.451819 | -0.424688 |
| H2 | -2.187049 | -1.606934 | -0.089180 |
| H3X | -0.949981 | 0.872402 | -2.817320 |
| H4 | -1.600715 | -1.245376 | -3.310749 |
| H51 | -2.927425 | -3.774689 | -3.342158 |
| H52 | -4.573508 | -3.717473 | -2.615175 |
| H6 | -5.274789 | -1.415948 | -1.383207 |
| H7 | -4.000849 | 0.733298 | -2.524891 |
| H9 | -4.807476 | -0.117872 | 1.094208 |
| H10 | -4.817797 | 1.924686 | 2.326243 |
| H111 | -3.566580 | 4.412341 | 2.287071 |
| H112 | -1.966711 | 4.439193 | 1.456637 |
| H12 | -1.328216 | 2.079558 | 0.137144 |
| H14 | -1.802662 | 0.580448 | 2.967283 |
| H15 | 0.444998 | -0.833550 | 2.028330 |
| C1A | 0.612632 | -1.637362 | -0.304263 |
| N2A | 1.834741 | -1.723280 | 0.620476 |
| C3A | 2.459528 | -2.871791 | 0.746124 |
| O3A | 2.059735 | -3.949978 | 0.095804 |
| C4A | 3.670567 | -3.013757 | 1.645627 |
| N5A | 4.235995 | -1.979983 | 2.411684 |
| C6A | 5.322003 | -2.470085 | 3.104507 |
| C7A | 5.469716 | -3.828085 | 2.792709 |
| C8A | 4.436684 | -4.170151 | 1.879527 |
| Br6A | 6.344012 | -1.382732 | 4.243712 |
| Br7A | 6.810410 | -4.969312 | 3.479735 |
| H1A1 | 1.061330 | -1.459378 | -1.281551 |
| H1A2 | 0.123469 | -2.608733 | -0.231760 |
| H2A | 2.115682 | -0.889226 | 1.102327 |
| H5A | 3.926197 | -1.015722 | 2.473439 |
| H8A | 4.254449 | -5.146550 | 1.430599 |
| C1B | 0.577512 | 1.292552 | 1.808972 |
| N2B | 1.002545 | 1.890009 | 0.508464 |
| C3B | 2.155464 | 1.478715 | -0.163229 |
| O3B | 2.878351 | 0.577192 | 0.256285 |
| C4B | 2.518953 | 2.177441 | -1.459668 |
| N5B | 1.782131 | 3.219417 | -2.050214 |
| C6B | 2.404181 | 3.610105 | -3.215829 |
| C7B | 3.550994 | 2.823927 | -3.391562 |
| C8B | 3.622922 | 1.926893 | -2.292097 |
| Br6B | 1.715132 | 4.984951 | -4.293299 |
| Br7B | 4.758215 | 2.959022 | -4.838512 |
| H1B1 | 0.087610 | 2.063470 | 2.403767 |
| H1B2 | 1.529803 | 1.034374 | 2.272207 |
| H2B | 0.458178 | 2.670537 | 0.125401 |
| H5B | 0.927223 | 3.642594 | -1.706311 |
| H8B | 4.389580 | 1.173391 | -2.111625 |

**Tubocurarine (4)**

| | | | |
|---|---|---|---|
| C1 | 0.837179 | -1.444167 | -3.070455 |
| N2 | 1.758805 | -2.058683 | -4.120049 |
| C3 | 1.316272 | -3.496275 | -4.448324 |
| C4 | -0.056078 | -3.503147 | -5.119821 |
| C5 | -1.037529 | -2.506479 | -4.521688 |
| C6 | -2.396002 | -2.537288 | -4.932564 |
| C7 | -3.314727 | -1.648866 | -4.414390 |
| C8 | -2.909009 | -0.679739 | -3.460850 |
| C9 | -1.551817 | -0.657466 | -3.054145 |
| C10 | -0.634512 | -1.563625 | -3.594658 |
| C11 | 1.789163 | -1.199324 | -5.385218 |
| C12 | 3.205019 | -2.129448 | -3.582266 |
| O13 | -4.647910 | -1.563265 | -4.716474 |
| C14 | -5.048278 | -1.753237 | -6.106469 |
| O15 | -3.767929 | 0.213435 | -2.919882 |
| O16 | -1.131790 | 0.261223 | -2.145839 |
| C17 | -1.465266 | 0.304364 | -0.798557 |
| C18 | -1.396084 | 1.565913 | -0.223905 |
| C19 | -1.854598 | -0.791078 | -0.032300 |
| C20 | -1.732947 | 1.737230 | 1.124277 |
| C21 | -2.169688 | -0.615418 | 1.320486 |
| C22 | -2.131847 | 0.649963 | 1.903575 |
| C23 | -2.495463 | 0.878020 | 3.363280 |
| C24 | -1.127312 | 0.707103 | 4.256148 |
| N25 | -1.536541 | 1.006531 | 5.678419 |
| C26 | -1.775832 | 2.465165 | 5.953456 |
| C27 | -0.634505 | 3.333286 | 5.606217 |
| C28 | 0.156735 | 2.901386 | 4.348941 |
| C29 | 1.160619 | 3.744340 | 3.851540 |
| C30 | 1.904069 | 3.368732 | 2.733458 |
| C31 | 1.668179 | 2.130693 | 2.132368 |
| C32 | 0.679226 | 1.294193 | 2.622287 |
| C33 | -0.066826 | 1.675216 | 3.762030 |
| C34 | -0.626579 | 0.378698 | 6.710564 |
| O35 | 2.916711 | 4.116440 | 2.172374 |
| C36 | 3.364976 | 5.288442 | 2.921671 |
| O37 | 2.460222 | 1.787312 | 1.009649 |
| C38 | 2.575541 | 0.422185 | 0.689367 |
| C39 | 1.757453 | -0.126688 | -0.267594 |
| C40 | 3.521893 | -0.353759 | 1.352731 |
| O41 | 4.299965 | 0.288517 | 2.281963 |
| C42 | 3.647066 | -1.693263 | 1.026802 |
| C43 | 2.806270 | -2.279362 | 0.062400 |
| C44 | 1.866777 | -1.481761 | -0.619175 |
| C45 | 0.944252 | -2.089617 | -1.692943 |
| H1 | 1.072131 | -0.389355 | -2.928083 |
| H3A | 2.060735 | -3.876812 | -5.147656 |
| H3B | 1.291659 | -4.101384 | -3.542047 |
| H4A | 0.096705 | -3.226337 | -6.162958 |
| H4B | -0.481669 | -4.504270 | -5.051077 |
| H6 | -2.718931 | -3.278785 | -5.663316 |
| H11A | 1.772239 | -1.881320 | -6.235333 |
| H11B | 0.903559 | -0.564339 | -5.360731 |
| H11C | 2.707036 | -0.612183 | -5.355648 |
| H12A | 3.367407 | -1.240012 | -2.973469 |
| H12B | 3.279577 | -3.040684 | -2.988811 |
| H12C | 3.875615 | -2.156450 | -4.441144 |
| H14A | -4.340058 | -2.444371 | -6.563479 |
| H14B | -6.056990 | -2.166260 | -6.103275 |
| H14C | -5.023304 | -0.778718 | -6.594106 |
| H15 | -4.177942 | -0.152566 | -2.197046 |
| H18 | -1.112618 | 2.408303 | -0.854878 |
| H19 | -1.912536 | -1.781246 | -0.484308 |
| H20 | -1.696035 | 2.747084 | 1.532851 |
| H21 | -2.502355 | -1.489155 | 1.880857 |
| H23A | -2.826422 | 1.901637 | 3.538704 |
| H23B | -3.212795 | 0.137039 | 3.716099 |
| H24 | -0.805268 | -0.316445 | 4.064475 |
| H25 | -2.425335 | 0.499823 | 5.842692 |
| H26A | -2.051635 | 2.556734 | 7.004002 |
| H26B | -2.627108 | 2.830932 | 5.379310 |
| H27A | -0.905504 | 4.389056 | 5.603180 |
| H27B | 0.113639 | 3.207688 | 6.388908 |
| H29 | 1.342714 | 4.708108 | 4.327031 |
| H32 | 0.507623 | 0.314627 | 2.176076 |
| H34A | -0.416830 | -0.643113 | 6.394324 |
| H34B | 0.282714 | 0.978498 | 6.749611 |
| H34C | -1.156291 | 0.399030 | 7.662978 |
| H36A | 2.488342 | 5.902242 | 3.128704 |
| H36B | 3.826934 | 4.937318 | 3.844388 |
| H36C | 4.083525 | 5.825456 | 2.302476 |
| H39 | 1.031002 | 0.485885 | -0.801564 |
| H41 | 5.026724 | 0.646153 | 1.870449 |
| H42 | 4.375278 | -2.324167 | 1.536475 |
| H43 | 2.931328 | -3.329093 | -0.203163 |
| H45A | -0.033557 | -1.848175 | -1.276175 |
| H45B | 0.978477 | -3.178479 | -1.729106 |

**Vincristine (5)**

| | | | |
|---|---|---|---|
| N1 | 2.194340 | -1.701936 | 1.341816 |
| C2 | 1.082000 | -2.523979 | 1.231127 |
| C3 | 1.481953 | -3.651177 | 0.466598 |
| C4 | 2.872912 | -3.438820 | 0.146227 |
| C5 | 3.835553 | -4.244180 | -0.580964 |
| C6 | 5.127633 | -3.760030 | -0.705905 |
| C7 | 5.529306 | -2.505055 | -0.110232 |
| C8 | 4.632041 | -1.723246 | 0.588153 |
| C9 | -0.170682 | -2.029077 | 1.945786 |
| C10 | -1.531905 | -2.799599 | 1.674680 |
| C11 | 0.959510 | -4.981028 | -0.076293 |
| C12 | -0.519399 | -5.409219 | -0.216700 |
| N13 | -1.403833 | -4.559435 | -1.084559 |
| C14 | -1.404892 | -3.090981 | -0.962120 |
| C15 | -2.194240 | -2.585643 | 0.252430 |
| C16 | -3.634746 | -3.162515 | 0.174623 |
| C17 | -3.767259 | -4.676243 | -0.107824 |
| C18 | -2.775342 | -5.084019 | -1.241080 |
| C19 | 0.163405 | -2.206281 | 3.423726 |
| O20 | 0.940727 | -1.425448 | 3.984501 |
| O21 | -0.418735 | -3.254075 | 4.119892 |
| C22 | 0.030532 | -3.360464 | 5.471323 |
| O23 | -3.511470 | -5.426975 | 1.085996 |
| C24 | -5.240332 | -4.920483 | -0.552980 |
| C25 | -5.691343 | -6.387782 | -0.716796 |
| C26 | -0.607303 | -0.601355 | 1.632666 |
| C27 | -0.310203 | -0.019774 | 0.388030 |
| C28 | -0.688595 | 1.317252 | 0.118234 |
| C29 | -1.429781 | 2.039872 | 1.043525 |
| C30 | -1.822768 | 1.463494 | 2.275306 |
| C31 | -1.381280 | 0.165385 | 2.576944 |
| O32 | -1.751112 | -0.365965 | 3.835638 |
| C33 | -3.136128 | -0.243432 | 4.163698 |
| N34 | -1.711799 | 3.306750 | 0.520883 |
| C35 | -2.458350 | 4.165478 | 1.177588 |
| O36 | -2.799815 | 5.299725 | 0.620787 |
| C37 | -1.129943 | 3.517052 | -0.831607 |
| C38 | -0.436427 | 2.143154 | -1.135040 |
| C39 | 0.989576 | 2.224915 | -1.662639 |
| C40 | 1.992485 | 3.184792 | -0.919069 |
| C41 | 1.192452 | 4.298682 | -0.143018 |
| C42 | -0.089531 | 4.717494 | -0.880469 |
| C43 | -1.174105 | 1.414660 | -2.337134 |
| C44 | -0.421370 | 1.867703 | -3.593447 |
| N45 | 0.752897 | 2.592341 | -3.066724 |
| C46 | 1.915730 | 2.780883 | -3.924733 |
| C47 | 2.933650 | 3.695187 | -3.244088 |
| C48 | 2.941271 | 3.858674 | -1.903448 |
| O49 | -0.653949 | 5.864257 | -0.272854 |
| C50 | 0.256998 | 5.130175 | -2.301361 |
| O51 | -0.510059 | 4.793682 | -3.207243 |
| O52 | 1.408449 | 5.865682 | -2.550551 |
| C53 | 1.530481 | 6.235772 | -3.925487 |
| O54 | 1.997309 | 5.476754 | 0.042285 |
| C55 | 1.708340 | 6.300867 | 1.123328 |
| O56 | 0.781075 | 6.153898 | 1.917988 |
| C57 | 2.629472 | 7.492376 | 1.335611 |
| C58 | 2.849063 | 2.449937 | 0.136748 |
| C59 | 3.994167 | 1.538391 | -0.387270 |
| C60 | 3.271410 | -2.215504 | 0.712112 |
| H1 | 2.188287 | -0.926053 | 1.809466 |
| H5 | 3.535575 | -5.196797 | -1.017579 |
| H6 | 5.862722 | -4.367563 | -1.233776 |
| H7 | 6.563480 | -2.176940 | -0.214752 |
| H8 | 4.920230 | -0.783652 | 1.059544 |
| H10A | -1.357719 | -3.866417 | 1.814889 |
| H10B | -2.249979 | -2.376589 | 2.377198 |
| H11A | 1.337356 | -5.717415 | 0.632979 |
| H11B | 1.414906 | -5.159985 | -1.050300 |
| H12A | -0.978572 | -5.316531 | 0.767510 |
| H12B | -0.601900 | -6.434519 | -0.577327 |
| H14A | -1.833922 | -2.698185 | -1.883936 |
| H14B | -0.370079 | -2.759110 | -0.877722 |
| H15 | -2.213815 | -1.496720 | 0.208121 |
| H16A | -4.141895 | -2.923183 | 1.109300 |
| H16B | -4.130896 | -2.638039 | -0.641992 |
| H18A | -3.143820 | -4.695162 | -2.190350 |
| H18B | -2.738011 | -6.172450 | -1.286056 |
| H22A | -0.704636 | -2.862105 | 6.103198 |
| H22B | 0.107006 | -4.419946 | 5.715762 |
| H22C | 1.001891 | -2.869787 | 5.532943 |
| H23 | -3.660093 | -4.904134 | 1.812753 |
| H24A | -5.896168 | -4.429933 | 0.166283 |
| H24B | -5.366507 | -4.420067 | -1.513065 |
| H25A | -5.425270 | -6.753270 | -1.708624 |
| H25B | -5.201187 | -7.012696 | 0.029749 |
| H25C | -6.770820 | -6.464760 | -0.586783 |
| H27 | 0.310642 | -0.545944 | -0.337089 |
| H30 | -2.361343 | 2.052541 | 3.017640 |
| H33A | -3.704612 | -0.322276 | 3.237032 |
| H33B | -3.382784 | -1.053274 | 4.850297 |
| H33C | -3.275552 | 0.730771 | 4.632301 |
| H35 | -2.839634 | 3.973298 | 2.180479 |
| H37 | -1.823164 | 3.839335 | -1.608573 |
| H39 | 1.603744 | 1.339281 | -1.499696 |
| H41 | 0.975935 | 3.819982 | 0.812004 |
| H43A | -1.054530 | 0.344659 | -2.167132 |
| H43B | -2.220516 | 1.719672 | -2.346750 |
| H44A | -0.030115 | 1.041595 | -4.187219 |
| H44B | -1.054010 | 2.507849 | -4.208329 |
| H46A | 1.555438 | 3.260734 | -4.834696 |
| H46B | 2.397125 | 1.831851 | -4.160734 |
| H47 | 3.656416 | 4.230736 | -3.859635 |
| H48 | 3.693861 | 4.526805 | -1.484748 |
| H49 | -1.339472 | 6.186666 | -0.773024 |
| H53A | 1.354548 | 5.340442 | -4.521751 |
| H53B | 0.781078 | 7.000398 | -4.130041 |
| H53C | 2.539281 | 6.620409 | -4.075414 |
| H57A | 2.621255 | 7.779253 | 2.387150 |
| H57B | 3.644729 | 7.226099 | 1.041588 |
| H57C | 2.287540 | 8.331447 | 0.729688 |
| H58A | 3.297920 | 3.191164 | 0.797966 |
| H58B | 2.191263 | 1.826393 | 0.742217 |
| H59A | 4.798384 | 2.157075 | -0.785473 |
| H59B | 4.377150 | 0.927940 | 0.430516 |
| H59C | 3.610737 | 0.890937 | -1.175867 |
